# Immunobiology of pulmonary fibrosis

**DOI:** 10.3389/fimmu.2025.1708716

**Published:** 2025-12-15

**Authors:** Samik Bindu, Hadida Yasmin, Uttam Barman, Subhradip Pandit, Khaled Masmoudi, Uday Kishore

**Affiliations:** 1 Department of Zoology, Cooch Behar Panchanan Barma University, Cooch Behar, West Bengal, India; 2 Department of Integrative Agriculture (CAVM), United Arab Emirates University, Al Ain, United Arab Emirates; 3 Department of Veterinary Medicine (CAVM), United Arab Emirates University, Al Ain, United Arab Emirates; 4 Zayed Centre for Health Sciences, UAE University, Al Ain, United Arab Emirates

**Keywords:** pulmonary fibrosis, innate immunity, alveolar macrophages, immunomodulation, fibroblasts, clinical trials

## Abstract

Pulmonary fibrosis, an interstitial lung disease, is characterized by progressive thickening and scarring of the lung tissue associated with shortness of breath, decreased vital capacity, and respiratory failure due to the inability to expand and contract the lungs during inspiration with severe morbidity and mortality. The median estimated survival is 2–5 years following diagnosis. Current understanding of how the disease initiates and progresses suggests a set of complex mechanisms involving genetic vulnerability, aging processes, and environmental factors. Mechanistically, the damage of the alveolar epithelial cells (AECs) (type I and type II), followed by recruitment of immune cells and transdifferentiation of fibroblast to myofibroblast play a crucial role in the initiation of a prolonged wound healing response in lungs which eventually leads to fibrosis if healing gets awry. Here, we have systematically reviewed the role of innate and adaptive immunity, as well as interactions of lung parenchyma cells together with the immune cells, cytokines, chemokines and other mediators in the context of pulmonary fibrosis. We have also discussed how the selection of pre-clinical models is important for understanding the disease and successful clinical trial outcome.

## 1 Introduction

Pulmonary fibrosis (PF) is a progressive, irreversible and lethal condition characterized by an overwhelming deposition of extracellular matrix leading to thickening and scarring of the lung tissue debilitating the lung function as manifested by decline in forced vital capacity (FVC) and diffusing capacity of carbon monoxide (DLCO). PF has increased significantly in the industrialized world (1). Epidemiological features suggest that multiple risk factors including aging and genetic factors, gender, environmental factors, particularly exposure to cigarette smoke, exposure to microorganisms, and unknown (idiopathic) causes may also contribute to the pathogenesis and persistence of the disease. The prevalence of idiopathic pulmonary fibrosis (IPF) is 7 to 1650 per 100,000 persons which is upto half of the total interstitial lung disease (ILD) cases (2, 3). 40,000 new cases are diagnosed each year in Europe validating the severity of the disease. The disease burden on society is massive considering the direct treatment cost of disease is around 25000 USD/person-year (2). Although PF is considered to be a disease confined to the lungs, its risk factors are shared by many comorbidities (eg. cardiovascular and degenerative diseases) (4).

Genetic vulnerability, aging processes, and environmental influences and their complex interaction trigger disease progression (5). PF is characterized by an excessive deposition of collagenous and non-collagenous extracellular matrix (ECM) components in the tissue or organ due to dysregulated wound repair systems (6). Wound healing response has four distinct phases, which starts with coagulation or clotting phase. It is then followed by inflammatory phase, which in turn, triggers fibroblast proliferation, migration and eventual trans-differentiation to myofibroblast, which is finally followed by tissue remodelling and restoration phase (7). After tissue damage, the epithelial and endothelial cells release pro-inflammatory mediators that trigger blood clotting in order to prevent blood loss. This triggers platelet aggregation, degranulation, vasodilation with increased permeability and subsequent infiltration of inflammatory immune cells {macrophages, neutrophils, monocytes, natural killer (NK) cells and dendritic cells (DC)} at the injured site (8). The role of monocytes, macrophages, neutrophils, cytokines, chemokines, inflammasomes, T cells and B cells in the disease pathology is very intricately regulated (9). During normal healing, myofibroblasts that form scar tissues are deactivated and undergo programmed cell death and eventually get cleared. On the contrary, during the pathogenesis, the myofibroblasts become persistence with overwhelming deposition of ECM causing thickening, scarring and fibrotic remodelling of the tissue affecting the tissue’s functional capacity (10). Thus, mechanistically, PF has a highly complex pathogenesis orchestrated by epithelial, endothelial, mesenchymal (fibroblast) and immune cells together with their secretion of pro-fibrotic mediators of which the role of TGF-β1 is well established (11). This review elaborates the intricate interplay of the immune and the non-immune cells in modulating the lungs microenvironment leading to the establishment of PF.

## 2 Role of epithelial, endothelial, and fibroblast cells in PF

**Figure 1:**
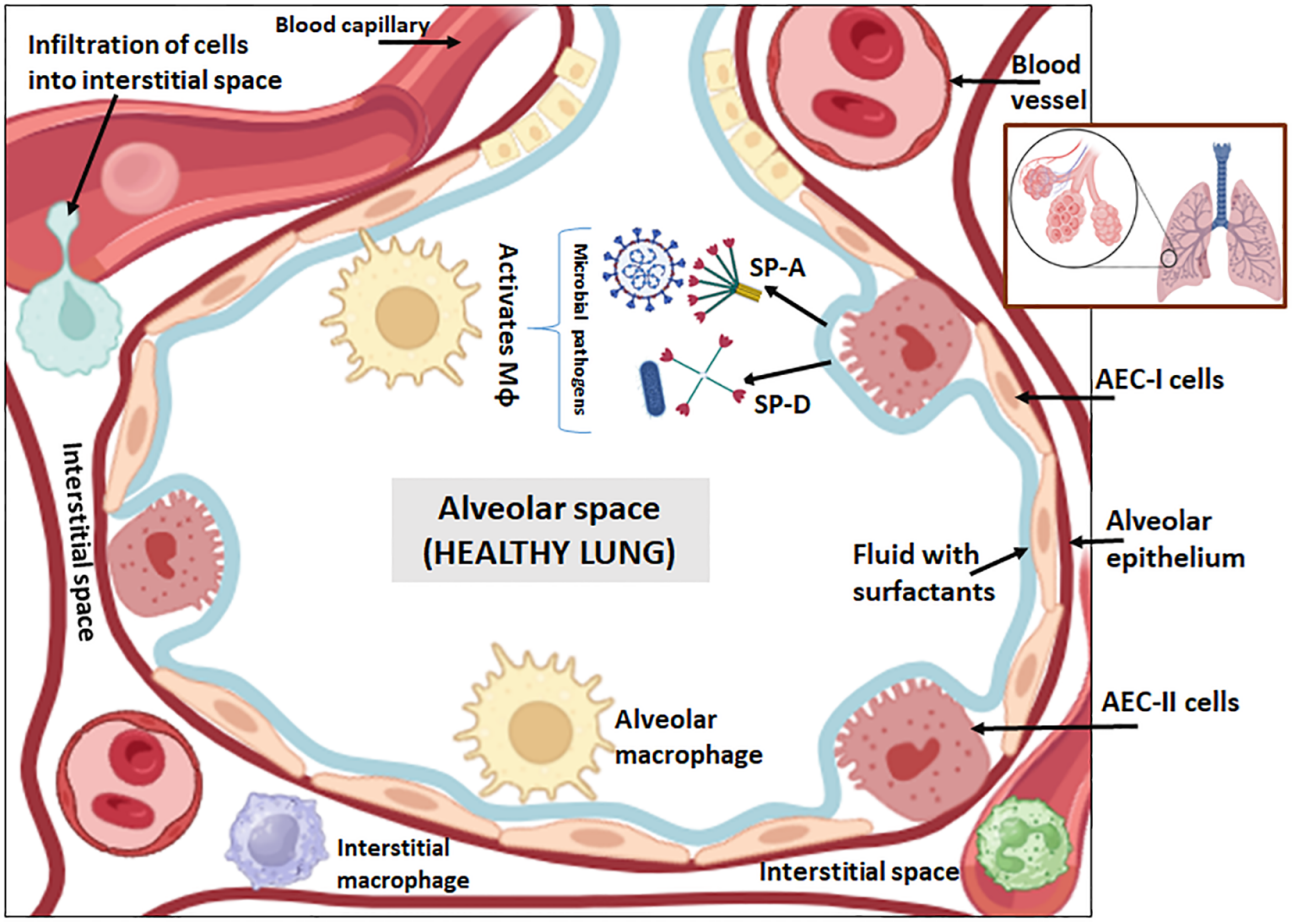
Cross sectional view of healthy lung alveoli. The alveolar epithelium is made up of type I alveolar epithelial cells (AEC-I) with squamous extensions and in between which are single cuboidal type II alveolar epithelial cells (AEC-II). The alveolar epithelium is covered by a fluid lining layer with surfactants which forms the surface film at the air-liquid interface. AEC-II consists of secretory organelles called lamellar bodies that stores surfactants proteins or collectins, i.e. SP-A, SP-B, SP-C and SP-D. The SP-A and SP-D has the capability to bind and neutralize wide group of microbial pathogens (bacteria, virus, fungi and parasites) and can modulate the immune response by activating alveolar macrophages for efficient clearance of invading pathogens at the mucosal surfaces of lungs.

The pathogenesis of PF involves a complex interaction between, epithelial cells, endothelial cells, fibroblasts and immune cells, associated with intricate intracellular signaling cascade (11). The pseudo-stratified airway epithelium of the lungs not only provides a mechanical barrier to the entry of foreign particles but also orchestrates innate as well as adaptive immunity (12). The number of basal cells dramatically falls as the alveolar space increases (12). Of these, alveolar epithelial cells type I (AEC-I) constitute 95% of the total surface area of the lung epithelium and perform gas exchange (13). The AEC-I cell lining imparts an impermeable barrier and keeps the alveolar air space dry by restricting fluid infiltration. AEC-I express a varied type of water and ion channels and tight junctions, which are potential targets of ROS and pro-inflammatory cytokines such as TGF-β, TNF-α, and IL-1β during PF (14, 15). AEC-II are the most abundant epithelial cells producing surfactant proteins, which enable gas exchange and act as progenitor cells for AEC-I (12) (**Figure 1****).** Surfactant system is mainly composed of phospholipids such as unsaturated phosphatidyl choline, dipalmitoyl phosphatidyl choline and neutral lipids. Besides, it also consists of surfactant-associated proteins termed surfactant proteins (SP), SP-A and SP-D that are large hydrophilic C-type lectins (collectins) which are important innate immune molecules while SP-B and SP-C are small, non-collagenous hydrophobic proteins, which participates in lowering the surface tension. Surfactant proteins are essential components of pulmonary homeostasis as they reduce the surface tension in the alveoli at the air-water interface, thus, preventing it from collapsing (16, 17). AEC-II cells can also trigger innate immunity in the lung through secretions of collectins, defensins and lysozymes (18). AEC-II cells secret SP-A and SP-D that have the capability to bind and neutralize a range of microbial pathogens (bacteria, virus, fungi and parasites) (19). SP-A and SP-D can modulate the immune response by activating alveolar macrophages for efficient clearance of pathogens (17) (**Figure 1****).** During tissue repairing and homeostasis, AEC-II cells act as progenitors and differentiate into AEC-I cells, and thus, replenish the alveolar epithelial barrier (20).

Depending on the environmental cues, AECs respond to injuries following one of the pathways such as apoptosis/necrosis, proliferation-transdifferentiation, re-epithelization or epithelial-to-mesenchymal transition (EMT) (21). The continuous and repeated microscopic injuries to AECs leading to dysregulated wound healing also accounts for the prolong preclinical phase of the PF (22). Studies have shown that bleomycin (BLM) treatment can trigger AECs injury, necrosis, apoptosis and which in turn, can trigger the production and activation of profibrotic markers such TGF-β, CTGF (connective tissue growth factor), and Shh (sonic hedgehog protein) (23, 24). These mediators trigger migration, proliferation, activation and fibroblast-to-myofibroblast transdifferentiation with eventual accumulation of ECM like collagen and elastic fibers (24). For example, one study has shown that BLM-induced DNA damage and activation of p53 lead to subsequent AEC-II to AEC-I transition state with senescence associated seceretory proteins (SASP) of which TGF-β1 plays a pivotal role in fibroblast-to-myofibroblast transdifferentiation (25). Another line of evidence supports the link between AEC apoptosis and PF. TGF-β1 acts as a potent inducer of AEC apoptosis via FAS-mediated pathways by downregulating p21 and activating caspase-8. Myofibroblasts are non-muscle contractile cells which play pivotal role in wound healing and are considered as the primary effector cells in fibrosis (26). During normal tissue restoration, myofibroblasts produce ECM components, and once the wound is resolved, they are eliminated through apoptosis (26). However, during chronic inflammation and injury, myofibroblasts become resistant to apoptosis, expressing senescent phenotype, leading to aberration in wound healing and overwhelming deposition of ECM, leading PF (26). In terms of cell types, fibroblasts are the most versatile mesenchymal cells. Whether a lung injury will resolve or worsen is determined in part by the fate of fibroblasts.

As per the recent lineage tracing experiments, the major sources of the fibrosis-associated myofibroblasts include mesenchymal lineages such as tissue resident fibroblasts and mesenchymal stem cells, bone marrow-derived circulating fibrocytes and lipofibroblasts (26, 27). Interestingly, in a recent study macrophage-myofibroblast transition has been implicated in an unilateral ureteral obstruction-induced PF (28). Contribution of macrophage-myofibroblast transition has also been shown in other fibrotic models, suggesting another possible source of myofibroblasts in the fibrotic milieu (29). Moreover, EMT and endothelial-to-mesenchymal transition (EndoMT) are also implicated as a source of myofibroblasts (30). Other than the EndoMT, a role of endothelial cells (EC) in PF are still underappreciated. In a seminal paper from Margaret Turner-Warwick in 1963, a correlation has been implicated in finger clubbing and change in vascular patterns in IPF patients (31). Whether the vascular abnormalities in IPF are a cause or effect is not clearly understood. On the other hand, due to the anatomical proximity of the AECs cells to the ECs, an injury to the alveoli possibly leading to vascular damage or remodeling cannot be excluded (32). In IPF, TGF-β1 secreted from damaged epithelial cells activate ECs leading to an imbalance of angiogenic and angiostatic factors such as vascular endothelial growth factor (VEGF), resulting in abnormal proliferation of ECs, with subsequent apoptosis. In addition, TGF-β1 from damaged epithelium and PDGF secreted from apoptotic ECs can trigger proliferation of vascular smooth muscle cells (VSMC) leading to arterial narrowing and eventually vascular regression (32). By secreting angiocrine factors such as cytokines, chemokines, ECM components and exosomes, ECs can regulate homeostasis and regenerative processes in the lungs (33, 34). ECs, by secreting connective tissue growth factor/CCN family member 2 (CTGF/CCN2), TGF-β1 and plasminogen activator inhibitor-1 (PAI-1), have been shown to recruit and activate fibroblasts to produce collagen during PF (35). Moreover, a role of ECs in recruiting macrophages to the injured site and contributing to the fibrotic milieu in the BLM-treated lungs of mice has also been reported (35). In the profibrotic role of ECs, factors such matrix metalloproteinase (MMP)-14, Jag1, endothelial transcription factor ETS-related gene (ERG) and signaling pathways such as Wnt/β-catenin, Notch and sphingosine-1-phosphate (SIP1) GPCRs have been implicated (32). Recently, by endothelial single cell RNA-sequencing, a number of transcription factors were identified such as small proteins and mothers against decapentaplegic (SMAD) family member 6 (SMAD6), forkhead box F1 (FOXF1), ETS Variant Transcription Factor 6 (ETV6) and lymphoid enhancer binding factor 1 (LEF1), of which, FOXF1 gained special attention. FOXF1 deficient ECs not only triggered human fibroblast proliferation, invasion and activation of myofibroblast, but simultaneously stimulated macrophage migration via secretion of IL-6, TNF-α and C-C motif chemokine ligand 2 (CCL2). Mechanistically, FOXF1 by positively regulating Rras gene promoter inhibited TNF-α and CCL2. Reduced FOXF1 was observed in endothelial cells in BLM-induced lungs of mice as well as lungs from IPF patients (32). ECs, via secretion of angiocrine factors, have also been reported to promote AEC-II transdifferentiation to AEC-I thereby inducing homeostasis in a damaged lung tissue. By targeting Fms-like tyrosine kinase 1 (Flt1), a VEGF receptor in the endothelial cells via delivery of microRNA-200c-3p can bring about AEC-II transdifferentiation and subsequent homeostasis (36). Thus, this emerging role of ECs in PF establishes them as more than bystander player. As a result, the complex interaction among the non-immune cells such as epithelial, endothelial and mesenchymal cells especially fibroblast-myofibroblast, in the background of repeated tissue injuries triggers the profibrotic signaling pathways which leads to thickening and scarring of the lung tissue resulting in PF. However, in this pathological background, immune cells are also implicated which requires a special discussion.

## 3 Immune response and pulmonary fibrosis

**Figure 2:**
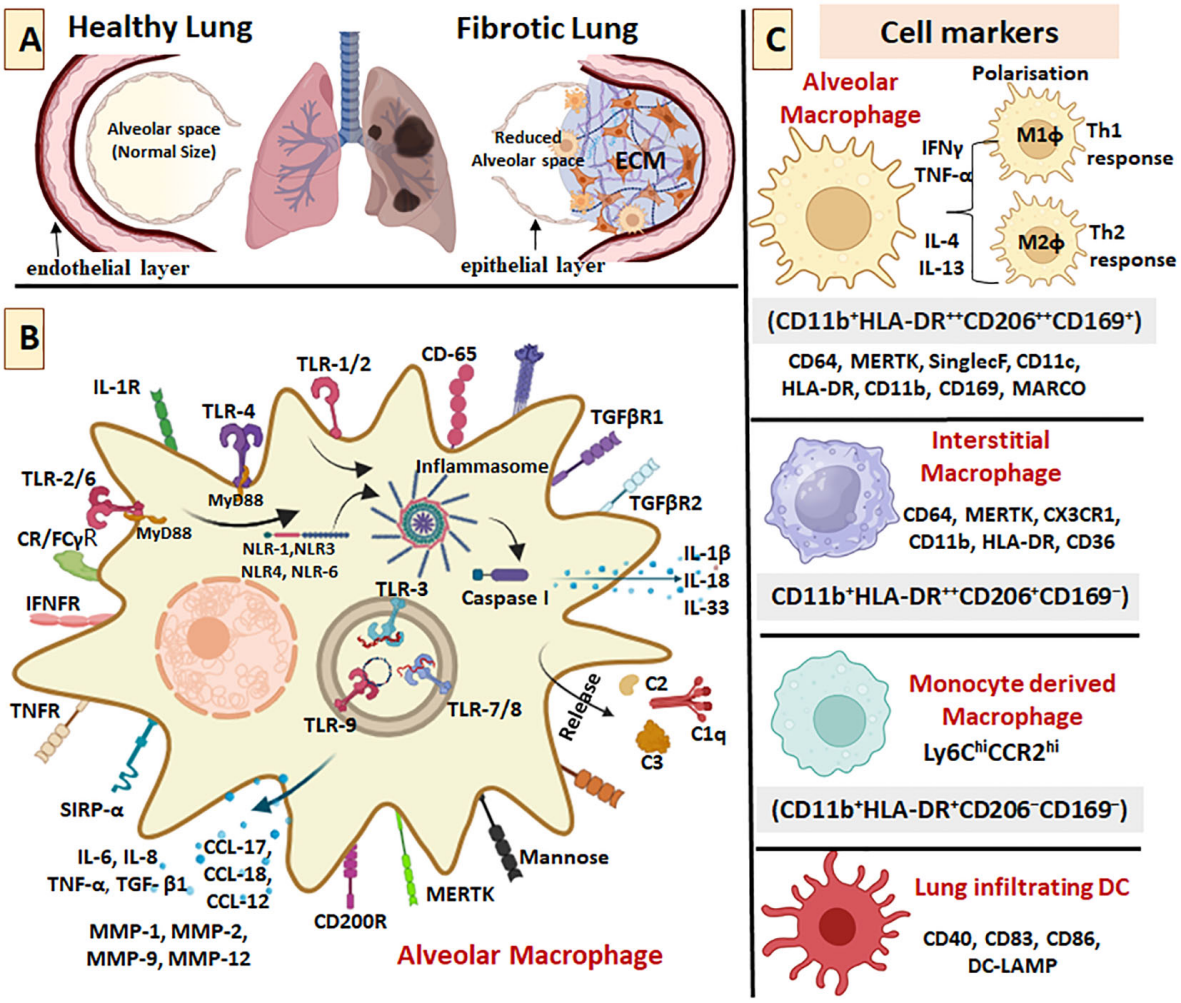
Different type of macrophages and its surface markers in lungs. **(A)** A healthy lung shows extended alveolar space for appropriate gas exchange, whereas in fibrotic lungs the alveolar space gets reduced due to the formation of extracellular matrix (ECM) in the alveolar layer extending to the interstitial space which leads to the loss of alveolar-capillary barrier basement membrane, thus, reducing the gas exchange surface. **(B)** Alveolar macrophage (AM) is one of the very vital immune cell, playing critical role in clearing the intracellular and extracellular pathogens invading the lungs. Some of the pertinent cell markers, cytokines, chemokines and enzymes are shown. **(C)** Both AMs and IMs undergoes polarization to M1 and M2 macrophages.The heterogeneity within AM populations in human can be screened through several markers, where AMs were CD11b^+^HLA-DR^++^CD206^++^CD169^+^, interstitial macrophages were CD11b^+^HLA-DR^++^CD206^+^CD169^−^ and monocytes were CD11b^+^HLA-DR^+^CD206^−^CD169^−^.

**Figure 3:**
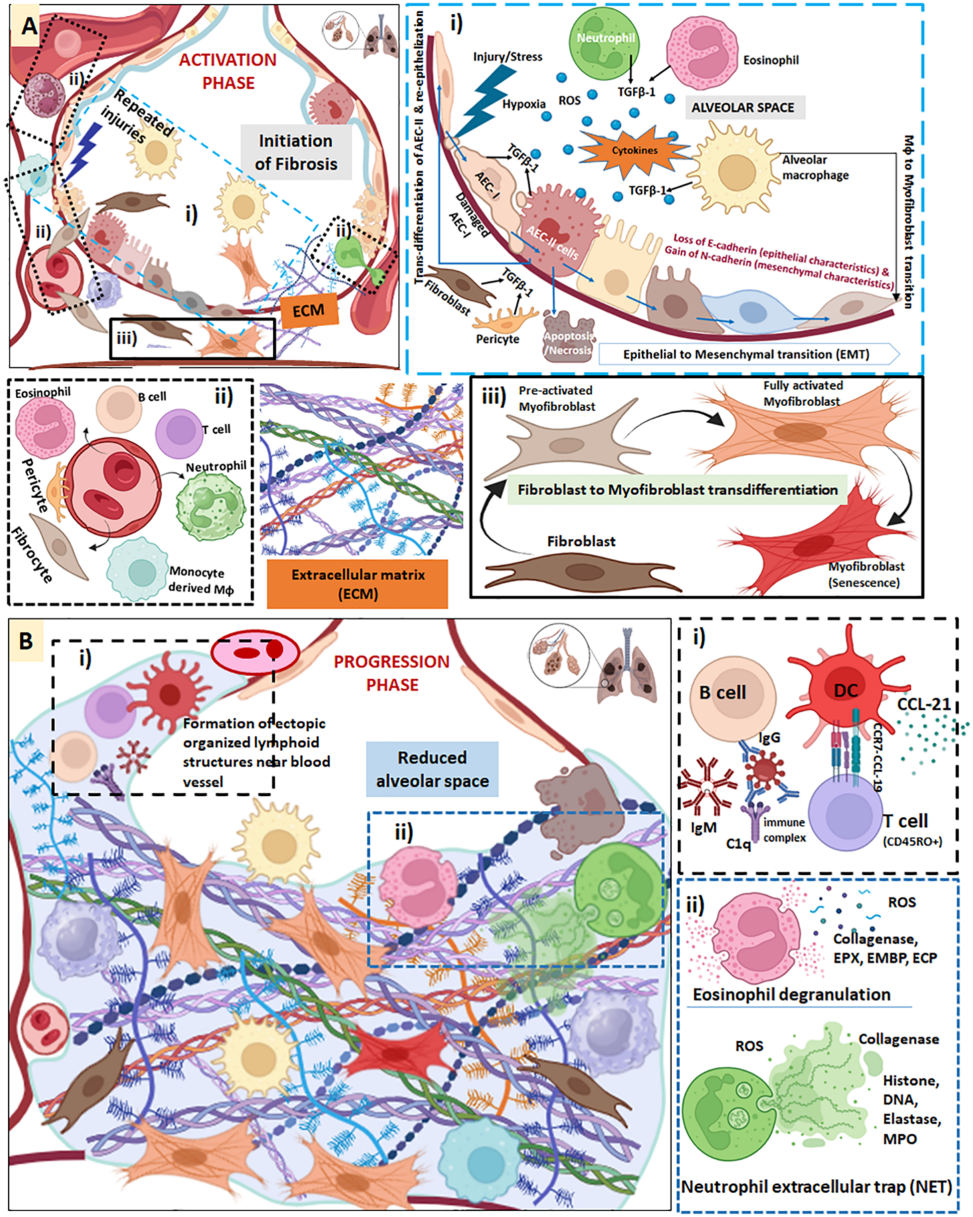
**(A)** Activation phase of lung fibrosis. **(i)** Several repeated injury of the alveolar epithelium due to assault by different factors leads to the initiation of PF. AEC-I undergoes apoptosis and are replaced by AEC-II. Both these cells secrete TGF-β1. The other cellular sources of TGF-β1 in lungs are AMs, bronchial epithelium, neutrophils, eosinophils, pericytes, fibroblasts and myofibroblasts. TGF-β1 triggers epithelial to mesenchymal transition (EMT) of AECs and a potent inducer of apoptosis in AECs. EMT is characterized by loss of epithelial characteristics (E-cadherin) and gain of mesenchymal features (N-cadherin). AM also undergoes differentiation to myofibroblasts. ROS is generated by macrophages (Mφ), neutrophils and eosinophils during inflammation activating several signaling pathways. **(ii)** Release of several chemoattractant factors and cytokines from the alveolar epithelium leads to infiltration of several cell types, such as monocytes, T lymphocytes, B lymphocytes, dendritic cells, neutrophil, eosinophil, pericytes and fibrocytes. All these infiltrating cells secretes several Th-1 mediators, thus, aggravating the inflammation in the lungs. **(iii)** Fibrocytes differentiates to fibroblasts and TGF-β1 triggers the fibroblast to myofibroblast transdifferentiation. Continuous secretion of TGF-β1 leads to abnormal activation of fibroblast and accelerates epithelial cell senescence. TGF-β1 is also capable of triggering senescence in activated fibroblasts/myofibroblast. The fibroblasts transdifferentiation is characterized by the synthesis of profibrotic proteins such as collagen, fibronectin and α-SMA eventually leading to the ECM deposition. **(B)** Progression phase of lung fibrosis. AMs and IMs gradually polarizes to its M2 phenotype, that mediates Th2 response and fibrosis continues. IL-4, IL-8, IL-13, IL-10 and IL-33 trigger M2 polarization of macrophages, which in turn promoted to a hyper-profibrotic phenotype by IL-4, IL-6 and IL-13. The fibrolytic macrophages facilitate ECM degradation by expressing MMPs such as MMP-1, MMP-2, MMP-14, MMP-2, MMP-9, MMP-13 and other secretory proteins and receptors such as cathepsin, integrin, mannose receptors, urokinase plasminogen activator receptor-associated protein (uPARP), milk fat globule epidermal growth factor 8 (Mfge8) Stabilin-1, hepatic growth factor (HGF) and several others. **(i)** CCL19, CXCL12, CCL21 contributes to DC recruitment and lymphoid neogenesis, forming ectopic organized lymphoid structures with activated non-proliferating B and T lymphocyte aggregates in lungs resembling lymphoid follicles. **(ii)** Eosinophils infiltrating the alveolar epithelium secretes enzymes, proinflammatory cytokines and generates ROS. Infiltrating neutrophils deploy neutrophil extracellular traps (NET) formation. All of these contributes to PF pathogenesis.

A healthy lung has an extended alveolar space for appropriate gas exchange, whereas in fibrotic lungs the alveolar space gets reduced due to the formation of ECM in the alveolar layer extending to the interstitial space which leads to the loss of alveolar-capillary barrier basement membrane, thus, reducing the gas exchange surface (**Figure 2A****).** Repeated injuries to the alveolar epithelium due to extrinsic and intrinsic factors (ageing, male sex, smoking, exposure to chemicals, environmental pollutants, viruses, genetic predisposition, and long-term exposure to certain drugs) lead to the initiation of PF. AEC-I undergoes apoptosis and are replaced by AEC-II; both these cells secrete TGF-β1. The other cellular sources of TGF-β1 in lungs are alveolar macrophages (AMs), bronchial epithelium, neutrophils, eosinophils, pericytes, fibroblasts and myofibroblasts (**Figure 3Ai****).** Reactive oxygen species (ROS) is generated by macrophages, neutrophils and eosinophils during inflammation activating several signaling pathways and thereby re-enforcing the tissue damage (**Figure 3Ai**). Epithelial damage and apoptosis trigger infiltration of immune cells initiating inflammation. Histopathological finding suggests immune cell infiltration in the lung parenchyma universally in all patients diagnosed with PF (37). Release of several chemoattractant factors and cytokines from the alveolar epithelium leads to infiltration of several cell types, such as monocytes, T lymphocytes, B lymphocytes, DCs, neutrophils, eosinophils, pericytes and fibrocytes. These infiltrating cells secrete Th1 cytokines, thus, aggravating the inflammation in the lungs (**Figure 3Aii**). Fibrocytes differentiate into fibroblasts and TGF-β1 triggers the fibroblast-to-myofibroblast transdifferentiation. Continuous secretion of TGF-β1 leads to abnormal activation of fibroblast and accelerates epithelial cell senescence. TGF-β1 is also capable of triggering senescence in activated fibroblasts/myofibroblasts. The fibroblast transdifferentiation is characterized by the synthesis of profibrotic proteins such as collagen, fibronectin and α-SMA eventually leading to the ECM deposition (**Figure 3Aiii**). Repeated injury keeps the wound healing prolonged, forming excessive ECM components making the lung tissue stiff (7). Apart from the role played by epithelial and fibroblast cells, both innate and adaptive immune mechanisms lead to the transition of robust lung inflammation to PF (38).

### 3.1 Innate immunity

Studies have implicated several innate immune cells such as macrophages, monocytes, neutrophils, eosinophils, mast cells, NK cells and DCs, and secreted chemokines and cytokines in the etiology of PF.

#### 3.1.1 Role of immune cells

##### 3.1.1.1 Monocytes and macrophages

Macrophages play a critical role in immune response and airway remodelling in PF. In terms of location in the lungs, macrophages are classically of two types; alveolar macrophages (AMs) located in alveolar and airway lumen, and interstitial macrophages (IMs) located in the lung parenchyma. AMs act as the natural gatekeepers within the lung alveoli, comprising of 90-95% of the cellular content (39–41). AMs are one of the most vital immune cells that play a critical role in clearing intracellular and extracellular pathogens invading the lungs. Here, some of the pertinent cell markers, cytokines, chemokines and enzymes assume great importance (**Figure 2B**). Several pattern recognition receptors (PRRs) such as toll like receptors (TLR)-1/2, TLR-2/6, TLR-4, TLR-3, TLR-7/8, TLR-9, NOD-like receptor (NLR)-1, NLR-3, NLR-4 and NLR-6, capable of binding to various pathogen-associated molecular patterns (PAMPs) or damage-associated molecular patterns (DAMPs) are present on/in AMs. PAMP-PRR engagement activates the macrophages to release different chemokines and cytokines to mount immune response through different pathways (**Figure 2B****).** Ontogenically, two AM populations exist in adult lung; i) tissue-resident AMs with self-renewal properties that develop shortly after birth and persist throughout the life span, ii) monocyte-derived AMs, which are recruited to lungs during injury and are derived from circulating monocytes (41). Interestingly, monocyte-derived AMs require an intermediate stage *i.e.* IM. Sequentially, repeated injuries/insults induce recruitment of blood circulating monocytes into the lung, where they first differentiate into intermediate IM and then undergo proliferative expansion, followed by migration into the alveolar space as AMs. The lung microenvironment provides continuous signals to maintain the AM pool in the lung. In both lung parenchyma and alveolar space, pulmonary macrophage reservoir is maintained as IM and self-renewable AMs respectively, further AMs are generated whenever required (42). Interestingly, both resident AMs and IMs, eventually get replaced by monocyte-derived AMs which also acquire self-renewal capacity similar to resident macrophages (43, 44). In the lung, AMs interact with alveolar epithelial cells and other immune cells and this co-ordinated interaction is mandatory for wound healing (45). Also, in lungs, AMs are the major sentinel cells recognizing PAMPs or opsonized pathogens through complement receptors (CR) or Fcγ receptors (FcγR) (46).

The heterogeneity within AM populations in humans can be gauged through several markers, as illustrated in **Figure 2C**. AMs and IMs express universal macrophage-markers such as MERTK (an efferocytic receptor tyrosine kinase) and CD64, which distinguish them from monocytes and DCs. AM-specific markers include Siglec F and CD11c while IM-specific markers include CD11b and CX3CR1 (47, 48). In human, HLA-DR and CD11b are common to both AM and IM, while CD169 and MARCO being specific to AM and CD36 being specific for IM. Both AMs and IMs are implicated in lung homeostasis. AM by secreting inflammatory regulators, such as CCL18, MGF-E8, FSP-1 and MMPs, stimulate fibroblasts and alter collagen synthesis (48). For IMs, there are two subsets: Lyve1^hi^ MHCII^lo^ and` Lyve1^lo^ MHC II^hi^ localized in perivascular area and alveolar interstitium, respectively, with an increased expression of the latter during fibrosis (44). In case of IMs, one study has linked the depletion of Lyve1^hi^ MHCII^lo^ IMs to the deterioration of PF (49). It is important to note that the AMs involved in the fibrosis are predominantly monocyte-derived rather than tissue-resident. Notably, the tissue-resident AMs undergo cell death due to BLM toxicity. Monocyte-derived IMs eventually get transformed into AMs replacing the dead tissue resident AMs (50). Evidently, while depletion of resident AMs do not affect BLM-mediated PF, monocyte-derived AMs persist for almost one year in lung even after the resolution of PF, implicating monocyte-derived AMs in PF compared to tissue-resident AMs (43). In the circulation two types of monocytes are present: Ly6C^hi^ CCR2^hi^monocytes (classical monocytes) and Ly6C^lo^ CCR2^lo^monocytes (non-classical), classical monocytes infiltrate the lungs in CCR2-dependent as well as independent manner during PF (43). In CCR2-deficient mice, PF was found to be reduced, likely due to impaired CCR2-dependent monocyte infiltration (43). In the lung, monocyte-derived AMs, recruited by the Fizz1 receptors of AEC-II, promote fibroblast activation and proliferation (43). Monocyte-derived AMs produce macrophage colony stimulating factor (M-CSF) for self-renewal and also secrete pro-fibrotic factors such as platelet-derived growth factor subunit A (PDGFA), arginase-1, matrix metallopeptidase 13 (MMP-13), VEGF and insulin-like growth factor 1(IGF-1) (43, 44). Recently, single cell transcriptomic analysis in an asbestos-induced murine PF model has shown Csf1 gene as a potential target in negatively regulating monocyte-derived AMs in the fibrotic niche (43, 51). In addition, global depletion of a Wnt co-receptor, named the low-density lipoprotein receptor-related protein 5 (Lrp5), showed resistance to BLM-induced PF in mice. Compared to *Lrp5*^+/+^ mice, in the fibrotic phase, the *Lrp5*^-/-^ mice showed a significantly-reduced monocyte-derived macrophages especially Siglec F^low^ AMs that are implicated in PF (52). Recent studies have shown that depletion of β-catenin in CD11c^+^ macrophages is associated with improved resolution of BLM-mediated fibrosis. Thus, activated Wnt/β-catenin signaling in macrophages has been linked to BLM and asbestos-induced PF (50). Single cell RNA sequencing using explanted lung samples revealed that AMs from fibrotic lungs had enhanced level of expression of genes such as IL1RN (encoding IL-1RA) and CHI3L1 (encoding chitinase-3-like-protein 1), which are pro-fibrotic in nature (53, 54). Macrophages have been reported to secrete TGF-β which plays a pivotal role in inducing PF along with other profibrotic mediators such as IL-1β and CCL18 (43, 44). In one study using IPF lung biopsies, an increase in *SPP1*^hi^ macrophages has been linked to PF (43). In addition, depletion of *Cx3cr1*^+^ in transitional macrophages has been linked to reduction in PF in BLM-induced model (43, 55). Macrophages that express Fra-2, an AP-1 transcription factor that targets Type VI collagen (ColVI), promote PF. However, Fra2/ColVI inactivation does not affect macrophage activation and recruitment, indicating a paracrine profibrotic control of macrophages by Fra2/ColVI (56).

As evident, heterogeneity within AMs population can be assessed based on markers (**Figure 2C**) (57). Besides the marker-based heterogeneity, the variation in the polarization of macrophages add to the complexity of the PF. In healthy human lung, CD206, which indicates M2 polarization, is ubiquitously present on macrophages (58). During the early inflammatory phase of pathogenic invasion or tissue damage in lungs, M1 macrophages are activated by enhanced Th1 cytokine, IFN-γ and TNF-α. On the other hand, Th2 cytokines (IL-4, IL-13) drives the anti-inflammatory M2 macrophages designed to resolve the inflammatory response and the wound. However, in the pathological conditions such as in IPF and interstitial lung disease (ILD), M2 macrophage population tends to be higher. During the disease progression, AM and IM polarize to pro-inflammatory M1 phenotype (classically activated) and to anti-inflammatory/pro-fibrotic M2 (alternately activated), respectively (47). M2 macrophages have been more closely associated with PF than M1 macrophages (43). M2 macrophages produce TGF-β1 triggering the fibroblast-to-myofibroblast trans-differentiation (**Figure 3Aiii****).** Moreover, M2 macrophage-secreted IL-4 and IL-10 also induce fibrosis (43). In a co-culture experiment with mouse lung epithelial cells, M2 macrophages have been found to trigger EMT. In addition, M2 macrophages can also promote myofibroblast differentiation (47) (**Figure 3Aii****).** When macrophages were pre-treated with TGF-β receptor inhibitor, LY2109761, blocked EMT, confirming the role of alveolar M2 macrophages in inducing EMT. Mice depleted for a GTPase, Rac2, have been shown to be devoid of M2 (macrophages) and also found to be resistant to BLM-induced PF. The susceptibility to BLM-induced fibrosis in Rac2^-/-^ mice can be restored when M2 macrophages are injected establishing a crucial relationship among integrin-driven Rac2 signaling axis in macrophages, macrophage differentiation and PF (54, 59). It is worth noting that M1/M2 classification often fails to explain the complex role played by macrophages in PF. Recent studies have classified macrophages on the basis of their specific functions in tissue fibrosis. A subset of macrophages that promote fibrosis in the early stages are called profibrotic macrophages while another subset at the resolution stage that prevents fibrosis are called anti-fibrotic macrophages (fibrolytic macrophages). The fibrolytic macrophages facilitate ECM degradation by expressing MMPs (MMP-1, MMP-14, MMP-2, MMP-9, MMP-13) and other secretory proteins and receptors such as cathepsin, integrin, mannose receptors, urokinase plasminogen activator receptor-associated protein (uPARP), milk fat globule epidermal growth factor 8 (Mfge8), Stabilin-1, hepatic growth factor (HGF) (60).

Thus, macrophages play a pivotal role in the initiation of wound healing and its eventual aberration leading to fibrosis. In this pathology, based on origin, monocyte-derived AMs contribute significantly, while based on polarization, M2 macrophages are predominant contributors to fibrogenesis. However, other proposed categories of fibrotic and anti-fibrotic macrophages are also implicated in the pathology.

##### 3.1.1.2 Neutrophils

Other than macrophages, neutrophils also play a significant role in the pathophysiology of PF. Chemotactic factors released from recruit neutrophils to the lungs during PF (61). Neutrophils account for 60% of total leukocytes in blood and predominantly reside in the pulmonary capillaries compared to systemic circulation. This enables the neutrophil recruitment to the lung tissue in response to injury or infection; while they defend lung tissue against extracellular pathogens, neutrophils can also stimulate inflammation and pro-fibrotic pathways in lungs (62). Neutrophils can deploy neutrophil extracellular trap (NET) formation, release of cytokines and chemokines (TGF-β1, IL-6, IL-17, CXCL4) exosomes, and autophagy to induce PF (62, 63). The cytotoxic components of NETs include myeloperoxidase (MPO) and the externalized histones with capability to stimulate lung fibroblasts, and promote proliferation and differentiation to myofibroblasts (63, 64) (**Figure 3Bii****).** The DNA component from NETs can induce lung epithelial damage through inflammasome activation and ferroptosis in alveolar epithelial cells while it triggers fibroblast-to-myofibroblast transdifferentiation via TLR9-miR-7-Smad2 signaling pathway (63, 65, 66). It has been found that peptidylarginine deiminase type 4 (PAD-4) mediated chromatin deprotonation, forming citrullinated histone 3 sets the initial stage for NET formation. In the bronchoalveolar lavage fluid (BALF) of IPF patients, elevated citrullinated histone 3, a marker of NET, has been observed (67). Thus, BLM-induced PF in PAD-4 knockout mice was found to be associated with reduced expression of profibrotic mediators such as elastin, fibronectin 1, CTGF, FGF2 and collagen type I (63). The externalized histones of NET then activate platelets, which in turn, secrete TGF-β1 which eventually antagonizes antifibrotic IL-27 derived from macrophages, thus aggravating PF (67). Moreover, neutrophil-secreted chemokines (CXCL8/IL-8) and cytokines (G-CSF) are increased in BALF of PF patients (68). Elastase, derived from NET is involved in collagen formation and TGF-β activation (69). Neutrophil elastase (NE), has been attributed to maintaining ECM turnover (70). Mice deficient in NE were found resistant to asbestos as well as BLM-induced PF, similar to mice treated with NE-inhibitor (71, 72). NE also induces fibroblast proliferation and its trans-differentiation to myofibroblast (73). Recently, an increase in the number of neutrophils in the BALF fluid has been included in the IPF diagnosis (74). Thus, it is evident that neutrophils can be a potential therapeutic target in PF.

##### 3.1.1.3 Eosinophils and mast cells

In PF, the number of mast cells are significantly increased, especially tryptase positive mast cells, with subsequent negative impact on the baseline lung function (FVC). Elevated activation of mast cells existing in high density are also correlated with higher number of fibroblastic foci (75–77). Tryptase plays an important role in establishing a crosstalk between mast cells and epithelial and fibroblast cells along with the alveolar ECM (78). Interestingly, mast-cell deficient WBB6F1-W/W^v^ (MCD) mice are resistant to BLM-induced fibrosis compared to its congenic control (WBB6F1-^+/+^). On the other hand, this resistance can be reversed when mast cells are re-introduced into MCD mice (79). Moreover, nintedanib, the FDA approved drug against PF, targets mast cells by inhibiting a recombinant stem cell factor (SCF), which plays a crucial role in mast cell sensitization- through the tyrosine kinase receptor C-kit (also referred to as CD117 or the SCF receptor) (75). As a therapeutic option, masitinib that blocks tyrosine receptor kinase C-kit can be used to prevent mast cell hypersensitivity in PF (80). In PF, the mast cells also secrete PGD2, histamine, and HETE (11,12,15-hydroxy-5,8,10,14-eicosatetraenoic acid) in the lung tissue and attract eosinophils. Eosinophils are capable of releasing free oxygen radicals, damaging the lung tissue. Eosinophils are involved in the initiation and progression of pulmonary inflammation releasing several cytotoxic proteins stored in their secretory granules such as eosinophil peroxidase (EPO), eosinophil major basic protein (EMBP) and eosinophil cationic protein (ECP) (81) (**Figure 3Bii****).** About 40-50% increase in BALF eosinophilia has been associated with IPF patients, making it as a potential marker of progressive lung diseases including IPF (82–84). In addition to IPF, eosinophil count is also elevated in PF associated with a collagen vascular disorder (PF-CVD) in BALF. When these patients were classified as per the severity of the disease (chronic stable, progressive and acute progressive), the eosinophil and ECP levels were significantly elevated in acute progressive group along with type III procollagen peptide and albumin in BALF compared to chronic stable and progressive groups (85).

A deeper insight into the role of mast cells and eosinophil in PF is needed to understand the disease pathology and its connection to chronic inflammation.

##### 3.1.1.4 Natural killer cells

NK cells contribute 10% of lymphocytes in the lung (86, 87). In the BALF of IPF patients, a reduced expression of NKG2D on NK, NKT and γδ T cells has been observed which appears to be in response to the elevated expression of TGF-β or stress-induced ligand MHC class I polypeptide-related sequence A (MICA) in those patients (86). Since PF is characterized by accumulation of senescent cells, there appears a role of NK cells in clearing the senescent cells. NK cells probably mediate their antifibrotic effect via IFN-γ secretion. Recently, IL-12 has been shown to induce IFN-γ secretion by NK cells that suppresses collagen and α-SMA formation in fibroblasts (88). However, exogenous recombinant IFN-γ (recombinant IFN-γ-1b) treatment failed to restore PF condition as evident from the randomized, placebo-control multicentered trial INSPIRE (International study of Survival outcomes in idiopathic pulmonary fibrosis with Interferon-gamma-1b) trial. Importantly, the trial was stopped as no effective outcome was noticeable in the interim analysis (86, 89). NK cells also might show their antifibrotic effect either by removing infection or by cytokine production thereby keeping lung inflammation in check so that it may not reach profibrotic condition. Recent studies indicate that impairment in NK cell recruitment to the IPF lung reduces the probability of the clearance of senescent cells, thereby delaying fibrosis resolution (87, 90). Notably, in hepatic fibrosis, NK cells were found to be protective by selectively destroying collagen producing stellate cells. However, no such evidence in PF has been reported yet. Therefore, further research on the role of NK cells would be important to expand the therapeutic spectrum against PF.

##### 3.1.1.5 Dendritic cells

DCs also contribute to the pathogenesis of PF (91). In a BLM-induced PF mice model increased infiltration of CD11c^+^/MHC class II^+^ DCs, including CD11b^hi^ monocyte-derived inflammatory DCs, has been observed during the fibrotic phase (92). In IPF lung, immature DCs accumulate in regions of fibrosis and epithelial hyperplasia as well as in BALF. The infiltration of fully mature DCs (expressing CD40, CD83, CD86, DC-lysosome associated membrane protein) along with non-proliferating T and B lymphocytes, has been observed in IPF lung contributing to the formation of ectopic organized lymphoid structures (91, 93) (**Figure 3Bi****).** Homeostatic chemokines, CCL19, CXCL12 and CCL21, bring about DC recruitment and lymphoid neogenesis via the activation of lymphotoxin, LTα1β2 (91). These ectopic lymphoid structures in lungs resemble lymphoid follicles with lymphocyte aggregates containing activated non-proliferating B and T cells and mature DCs forming nearby blood vessels. T lymphocytes in these lymphoid structures are CD45RO^+^ memory phenotype and express CD40L (91, 94). CCL-19 is an essential factor for the lymphoid neogenesis and it acts as a ligand for T lymphocytes for binding to CCR7 of DCs (91) (**Figure 3Bi****).** In another recent study, compared to control, significantly increased accumulation of CD11b^+^ DC and CD103^+^ DC has been observed in mice where fibrosis is triggered with adenovirus-mediated TGF-β1 (AdTGF-β1) (95). Inhibition of TGF-β1 correlates with decreased expression of CD11c^+^CD103^+^ DCs, suggesting an anti-fibrotic role of these DC subsets (96). A co-culture experiment with lung fibroblasts (control and IPF) and DC cell line (MUTZ-3) confirmed the ability of the lung fibroblast to regulate the activation and maturation of DCs. This ascertains the ability of lung fibroblast in sustaining chronic inflammation in fibrotic lung by maintaining a pool of immature DCs *in situ* (97). DCs play an important role in fibrosis progression. A significant increase in DC count was observed in BLM-induced fibrotic murine lung. However, when the immunostimulatory effect of DCs was suppressed by an inhibitor VAG539 (a low molecular weight molecule with a capacity to bind with aryl hydrocarbon receptor on DC), fibrosis was ameliorated (98). A diphtheria toxin (DT) pre-treated DC depleted CD11c-DT receptor (DTR)-transgenic mice showed reduced fibrosis when exposed to BLM (98). However, both studies have shortcomings. Since VAG539 exerts its effect through binding with transcription factor aryl hydrocarbon receptor expressed in many cell types other than DC, it is not clear where the inhibitory effect is only DC-mediated. Moreover, as CD11c is also expressed in macrophages, it is hard to pinpoint the role of DC depletion after DT injection as the pre-requisite for reduced fibrosis in BLM treated mice (98). Thus, the relevance of BLM-induced mice model to human disease has also been questioned (98). Another recent report has shown that FMS-like tyrosine kinase-3 ligand (Flt3L), which is required for DC maturation, is up-regulated in IPF lung tissues as well as serum, indicating a possible accumulation of lung DCs during fibrogenesis (99). Flt3L deficient mice (Flt3L Knockout mice) with subsequent DC depletion develop more severe PF. On the contrary, mice with Flt3L supplementation with subsequent increase in DC count developed less severe PF, indicating a regulatory role of DCs in fibrosis. Although the role of Flt3L seems controversial, its pro- or anti-fibrotic properties may be dependent on the disease context *i.e.* the stage of disease progression or variation in the microenvironment. In a mouse model, when DTR (transgenic expression of a diphtheria toxin receptor) was expressed under the control of DC-specific promoter and the mouse was made DC depleted by DT treatment it showed more compromised lung function and severe PF. Various studies, thus, appear to suggest a negatively regulatory role of DCs in mouse PF model (98).

#### 3.1.2 Chemokines, cytokines and growth factors

Various cytokines and chemokines contribute significantly to the pathogenesis of PF (7, 100–103). The immune, epithelial, endothelial and fibroblast cells are not only the target of cytokines and chemokines during the aberrant wound healing response leading to PF, but they themselves secrete several cytokines and chemokines to orchestrate the pro-fibrotic signals.

Chemokines, subclassified into four subfamilies, CC, CXC, CX3C and XC chemokines, exert their biological activities by interacting with target-cell surface receptors such as CCR, CXCR, CX3CR and XCRs, respectively (8). The role of CX3C and XC in PF is very scarce (8). However, CC and CXC chemokines have been implicated in the pathogenesis of PF (8). CCL-1 (human: I-309; mouse: TCA-3) has been found to be enhanced in fibrotic lung tissues. CCL1 interacts with autocrine motility factor receptor (AMFR) and eventually triggers AMFR-Spry1mediated RAS-ERK-p70S6K signaling axis that ultimately elevates pro-fibrotic protein synthesis in fibroblasts (104). CCL2, or monocyte chemoattractant protein-1 (MCP-1) and its receptor CCR2, have been positively correlated with PF (105, 106), as evident from two-fold increase of CCL2 in BLM-induced fibrosis; higher level of CCL2 in BALF (154.3 pg/ml and 427.2 pg/ml) has been observed for surviving and non-surviving patients, respectively (8, 107). In another study using data from 17 IPF patients, although with lower values, similar results (68.65 pg/ml in IPF vs 4.56 pg/ml in control) were reported with increased CCL2 in IPF vs control (108). The variation in concentration from these two different studies may be due to variability in patient’s response and disease stage. A negative correlation has been shown between CCL2 and DLCO in IPF (DLCO 52.2 ± 13.9) patients vs control (DLCO 98.3 ± 10.3) (108). At the cellular level, normal lung macrophages, endothelial cells and smooth muscle cells have been also shown to express CCL2 along with AECs and fibroblasts; however, in IPF lungs, an enhanced CCL2 expression is probably contributed by AECs and fibroblasts (8). Furthermore, CCL2 upregulates endogenous TGF-β1 in fibroblasts thereby enhancing ECM production (8). Several factors have been attributed to the synthesis of CCL2 in fibroblasts: nuclear factor κB (NF-κB) and activator protein-1 (AP-1), thymic stromal lymphopoietin (TSLP)-TSLP receptor and STAT3 (8). Macrophage colony-stimulating factor (M-CSF), IL-13 or collagen type I also trigger CCL2 expression, thus promoting fibrosis. However, pulmonary CCR2^+^ CD4^+^ T cells can attenuate PF progression, indicating that in fibrosis, variation in CCR2 expression could have different effects on different subsets of immune cells (109). The observation that deletion of CCR2 protected mice from BLM-induced lung fibrosis makes CCR2 a potential drug target (110). Recently, in a phase II clinical trial using Carlumab, a CCL2 neutralizing antibody (humanized), failed to restore PF, probably due to an overstimulation of compensatory mechanisms in response to CCL2 neutralization (111).

Several reports have implicated CCL3 [macrophage inflammatory protein (MIP)-1α], CCL4 (MIP-1β) and CCL5 (RANTES) in the PF pathogenesis (8). Notably, one of the common receptors for these three chemokines is CCR5. In addition to the fibrotic lungs of IPF patients, CCL3 and CCL4 have also been found to be expressed in BLM, silica and mustard gas induced fibrotic lungs (8). CCL3 level was found slightly higher (1.73 pg/ml) than control (1.22 pg/ml) but statistically significant. In another study, between surviving and non-surviving patients the CCL3 level in BALF did not differ much. This could be summarized as difference between IPF vs control in both the studies that exhibit a narrow margin. BALF level of CCL4 has been moderately high (7.03 pg/ml) compared to control (3.10 pg/ml) in IPF (108). However, contradictory reports have shown that no correlation exists between BALF concentration of CCL3 and CCL4 with lung function of IPF patients (8). Probably, these mediators contribute to fibrotic pathology but changes in their expression level as in BALF may not have any significant effect on the PF pathogenesis. From the therapeutic perspective, one report has shown that anti-CCL3 antibody treatment restores BLM-induced PF (8, 112, 113). In a BLM-treated CCL3^-/-^ and CCR5^-/-^ mice (double KO mice), collagen deposition, intra-pulmonary macrophage abundance and fibroblast accumulation were found to be attenuated (114). CCL5, secreted by lung epithelial cells, is elevated in IPF patients, which is linked to eosinophil recruitment in the lungs (8, 115). This is evident as CCL5 upregulation is correlated with increased recovery of lymphocytes or eosinophils in BALF in IPF patients (115). In line with its ligands, CCR5 is expressed in fibroblast as well as AMs and lymphocytes. Thus, CCR5, being the receptor of potential pro-fibrotic chemokines (CCL3, CCL4 and CCL5), could act as a drug target in the IPF management (8). A global transcriptomic analysis of fibroblast from IPF patients has revealed a 22.8-fold upregulation of CCL8 (monocyte chemotactic protein, MCP-2), compared to fibroblasts derived from healthy control (8, 116). BALF CCL8 was also significantly higher in IPF patients compared to healthy control and is inversely correlated with DLCO/alveolar volume (VA) (85.6% vs control 57.0%). This projects CCL8 as a promising diagnostic and prognostic marker with 2.29 pg/ml in BALF as the cut-off for diagnosis of IPF (8, 116). IPF patients with higher CCL8 value (>28.61 pg/ml in BALF) showed a decreased survival rate. Furthermore, increased expression of two other chemokines, such as CCL17 (thymus and activation-regulated chemokine) and CCL22 (macrophage derived chemokine) and their shared receptor CCR4, has been associated with BLM-induced PF (8). Higher concentration of CCL17 (approx. 12 pg/ml) and CCL22 (approx. 75 pg/ml) are observed in BALF of IPF patients compared to healthy volunteers, which is also considered as a likely prognostic marker for the deterioration of conditions of IPF patients (8, 117). Moreover, CCL17 has recently been shown to activate fibroblasts and trigger the fibrotic cascade via TGF-β/SMAD signaling (118). In IPF patients, an inverse correlation has been found between CCL22 levels in BALF and DLCO/alveolar ventilation per minute (*V_A_*) values, re-enforcing the role of CCL22 in deterioration of lung function. However, despite a tendency of inverse correlation between CCL17 and DLCO/*V_A_* values, no statistical significance has been observed (117).

Mechanistically, CCL22 can recruit and activate CCR4^+^AMs in the lungs of IPF patients leading to lung dysfunction (8, 117). CCL1-mediated activation of CCR4 in macrophages is linked to PF in BLM-induced model (8, 119). Other than the macrophages, CD4^+^T cells in BAL of oropharyngeal flora (OPF) patients showed elevated level of CCR4. However, inconsistent with this finding, one group has shown a positive correlation between increased ratio of CCR4^+^ CD4^+^ T cell to CCR6^+^ CD4^+^ T cells to lung function preservation in IPF patients (119). This could be due to variation in the protective role of specific subsets of cells in IPF. Thus, this elusive nature of results deserves further scientific exploration. However, chemokines that determine the recruitment of protective T cells compared to detrimental T cells could be a therapeutic target (120). Expression of another important chemokine, CCL18, has been found in the BALF (6 ng/ml vs 1 ng/ml) and serum (149 ng/ml vs 39 ng/ml) of IPF patient vs control (8). Moreover, single nucleotide polymorphism (SNP) in the CCL18 gene predisposes IPF patient with poor prognosis (121). However, studies with opposite outcome have revealed no such correlation between CCL18 concentration and IPF disease progression. Nevertheless, these contradictory results have been attributed to study design, leaving the role of CCL18 still pertinent in PF (8). A cut-off of >150 ng/ml of serum CCL18 is considered indicative of mortality of IPF patients and is also positively correlated with decreased FVC and DLCO (122). AM synthesized CCL18 has been reported to induce collagen production from fibroblast independent of TGF-β1, which in turn, triggers CCL18 production, making it a vicious cycle. When AMs are exposed to collagen type I, it stimulates Akt phosphorylation through CD204, thereby converting AMs to alternatively activated macrophages (AAM) along with enhancing the production of CCL18. The CCL18 interacts with fibroblasts and triggers further collagen production through ERK-Smad3-Sp1 pathway (8, 123, 124). Next, CCL21 (6-Ckine, exodus-2 and secondary lymphoid-tissue chemokine) and its receptor CCR7 have been associated with the progression of PF, as evident from increased CCR7 expression in surgical lung biopsies. When exposed to CCL21, the IPF surgical lung biopsy-derived fibroblasts showed proliferative and migratory response. In addition, CCL21 via interaction with CCR7, triggers CCL5 production in IPF fibroblasts. CCL21 is also documented to alter the phosphorylation status of mitogen activated protein kinase 1/2, extracellular signal-regulated kinase ½ and ribosomal S6 kinase (90 kDa) in IPF fibroblasts (8, 125). All these CCL21 mediated pathways are inhibited by pertussis toxin (PTX), CCR7 neutralizing antibody, or siRNA against CCR7 (8, 125). Another result of immunoneutralization of CCR7 or CCL21 has been shown to reduce pulmonary remodelling in severe combined immunodeficiency CB-17-SCID/bg mice which has been intravenously pre-treated with IPF fibroblasts. Another chemokine, CCL24 (eotaxin -2) and its receptor CCR3, has been implicated in IPF pathogenesis. CCL24 level is increased in BALF from IPF patients *in vitro.* CCL24 triggers fibroblast proliferation and collagen synthesis (8, 126). In experimental PF model, treatment with anti-CCL24 monoclonal antibody, CM-101, attenuated collagen deposition and infiltration of immune cells in BALF, indicating a possible role of CCL24 in fibrogenesis (8, 127). Thus, despite some contradictory findings, it is evident that targeting the chemokines could be an attractive therapeutic approach in combating PF.

#### 3.1.3 Cytokines and growth factors

Various cytokines contribute to the immunomodulation and pathogenesis of PF (128); TNF-α and TGF-β1 deserve special mention, because they show synergistic effects in promoting inflammation, fibroblast activation, and directly regulate fibrotic pathways. An overlap between the actions of TNF-α and TGF-β1 have been proposed. One study has proposed TNF-α to be responsible for the regulation of TGF-β1 expression in Swiss 3T3 fibroblasts by inducing AP-1 transcription factor that transcriptionally upregulates TGF-β1 (129). TNF-α also appears to contribute to the PF by driving trans-differentiation of lung resident mesenchymal stem cells into myofibroblasts through NF-κB signaling pathway accompanied by β-catenin expression (130). Surprisingly, TNF-α transgenic mice are resistant to BLM-induced PF as compared to their transgene-negative littermates (131). TNF-α transgenic mice showed prostaglandin E2 upregulation and TNF receptor I downregulation. These opposite results could be due to differential effect of the endogenous (PF promoting) and exogenous (PF inhibiting) TNF-α (131). In BLM-challenged mice with PF, pulmonary delivery of TNF-α can lead to the resolution of the disease condition probably by reducing the numbers as well as programming status of pro-fibrotic macrophages (132). However, TNF-α neutralization with Etanercept did not bring any clinical benefit to IPF individuals (133). This could be due to several reasons including the complex signaling pathways that TNF-α unleashes, the use of surrogate endpoints and disease specific factors. From the available data of donated human lungs, it is evident that human lung responds consistently to repeated TGF-β1 exposure with consistent changes in fibrosis-linked genes following TGF-β1 treatment (134). Over-expression of TGF-β1, in mouse lungs, or adenoviral–mediated gene transfer of active TGF-β1 has been known to induce PF (135). This is hard to examine in gene deficient mice since TGF-β1 knockout mice cannot survive to adulthood and die shortly after birth (136). TGF-β1 mediates its action through its cognate receptors TGF-β1RII and TGF-β1RI, and finally exerts the downstream action through Smad proteins. At one hand, TGF-β1 triggers fibroblast-to-myofibroblast trans-differentiation while at the other hand, EMT of AECs (**Figure 3A**). As mentioned earlier, TGF-β1 is also a potent inducer of apoptosis in AECs while it triggers senescence in activated fibroblasts. Adhesion dependent FAK (focal adhesion kinase) acts as a switch, which determines the fate of ACEs towards EMT or apoptosis (5). During EMT, TGF-β1 activates transcriptional repressors such as zinc finger E-box binding homeobox 1 (ZEB1 and ZEB2) and Snail family transcription factors (SNAI1 and SNAI2), which are responsible for the loss of epithelial cell junction and apical-basal polarity (137). TGF-β1 triggers the synthesis of the extra type III domain A (EDA) containing fibronectin which precedes α-SMA formation and is a pre-requisite for fibroblast-to-myofibroblast transition (135). TGF- β1 also prevents the breakdown of ECM by not only controlling the expression of MMPs and plasminogen activators, but also by upregulating the synthesis of MMP-inhibitors and plasminogen activator-inhibitors (135). TGF-β1 can also trigger ROS (hydrogen peroxide) to induce signaling leading to myofibroblast formation, contractility and ECM deposition via NADPH oxidase isoform 4, NOX-4 (138). Moreover, TGF-β1 has been linked to metabolic reprogramming of the lung myofibroblasts during PF. Since PF is an aging-associated disease, an increase in TGF-β1 has been found in ageing human and mouse serum as well as in lung tissues (5, 139).

From a clinical perspective, interleukin (IL) level is also a good indicator of the extent of inflammation and poor prognosis in PF, and thus can be used to trace the progression and severity of the disease (128). For example, while IL-8 level in lung has a negative correlation with lung function, its level in serum of IPF patients determines the degree of neutrophilic alveolitis and its level in plasma is correlated with survival rate of the IPF patients (128). In IPF patients, an enhanced expression of IL-1β (2.23 fold), IL-8 (>10 fold), IL-17 A (9.67 fold) and IL-33 (3.59 fold) in BALF and IL-2 (10 fold), IL-8 (> 10 fold), IL-10 (10.12 fold), IL-12 (6.92 fold) in serum was found as compared to healthy control (128). Interestingly, IL level often varies between different stages of PF; for example, while IL-6 and IL-9 increases in IPF patients with acute exacerbation, no such increase is associated with stable IPF patients (128). IL-13, under the regulatory control of IL-33 and thymic stromal lymphopoietin (TSL), stimulates its downstream factors such as CCL2, and aggravates PF by disturbing the epithelial wound healing process (128). IL-8 promotes the proliferation and migration of mesenchymal stem cells (128). Moreover, IL-8, along with IL-4, IL-13, IL-10 and IL-33, induces M2 polarization of macrophages, which in turn, promotes a hyper-profibrotic phenotype by IL-4, IL-6 and IL-13 (128).

In BLM-treated mice, IL-1β facilitates neutrophil and lymphocyte recruitment to the damaged pulmonary tissue while IL-5 in the damaged lung recruits eosinophil thereby promoting PF (128). IL-10 not only improves the survival rate and reverses the weight loss in BLM-treated mice, but it also exhibits an anti-fibrotic effect by preventing downregulation of IFN-γ and upregulation of TGF-β1 (128). IL-17, a pro-inflammatory cytokine, promotes fibrosis by inhibiting autophagy as well as autophagy-induced cell death in BLM-induced PF model; thus IL-17 neutralizing antibody can improve survival of BLM-inflicted mice (128, 140). Moreover, IL-17A expression in BLM-induced PF model enhanced the expression of other known pro-inflammatory cytokines/chemokines such as TNF-α, IL-1, IL-6 and TGF-β, IL-8, CCL2, CXCL1 and CXCL5 by epithelial and endothelial cells (128, 141). Exogenous IL-17 can promote fibroblast proliferation and production of profibrotic proteins (141). Mechanistically, it stimulates fibroblasts via activation of NF-κB/Act1 signaling pathway (141). Dysregulated microbiota can also stimulate the expression of IL-17B, which by interacting with TNF-α can stimulate the secretion of neutrophil recruiting and Th17-cell promoting genes in BLM-induced PF mice (141). Another report has suggested a role of IL-17E (IL-25) and its receptor IL-RB in driving EMT of AECs and recruiting and activating lung fibroblast in IPF patients (141). Interestingly, IL-18, although a pro-inflammatory cytokine, can trigger a non-inflammatory pathway leading to PF via inhibition of anti-senescence protein Klotho, and thus, promoting senescence of lung fibroblast which adds to the pathogenesis (128, 142). In addition, it also triggers PF by downregulating epithelial features (E-cadherin) and upregulating α-SMA and SNAIL (128, 142). As opposed to the action of IL-18, intranasal instillation of IL-37, an anti-inflammatory cytokine, promotes anti-fibrotic effect in BLM-treated mice by upregulating LC3II (lipidated form of microtubule-associated protein 1 light chain 3), an autophagy activation marker, and thus, stimulating autophagy in fibroblasts. In addition, IL-37, which is normally expressed in AECs and AMs in healthy control is associated with reduced TGF-β1-mediated fibroblast proliferation and downregulation of TGF-β1 signaling pathway. However, in IPF patients and mouse model, IL-37 is reduced (128, 143). In case of IL-6, which has both pro-and anti-inflammatory role, it has been negatively correlated with lung function during PF. In the BLM-induced lung fibrotic milieu, M2 macrophages secrete IL-6, which along with IL-4 and IL-13, facilitates the production of macrophages with hyper-fibrotic phenotype, which aggravates PF by inducing ECM (128, 144). Macrophages through protease mediated cleavage produces soluble sIL-6Rα that stimulates IL-6 trans-signaling in murine PF and IPF patients (128, 144).

Increased expression of IL-6 and IL-33 in blood can synergistically affect the metabolism of whole body and cause muscle atrophy probably via activation of STAT3 and AMPK pathways in BLM-induced PF model (145). IL-33 can facilitate profibrotic M2 macrophage production leading to increased expression of profibrotic cytokines such as MCP-1, IL-6 and TGF-β1. Since IL-33 binds to its receptor suppression of tumorigenicity 2 (ST2) expressing immune cells, depletion of ST2 prevents mice from BLM-induced PF, validating a role of IL-33 in fibrogenesis (146). However, a bidirectional role has been suggested for IL-6; in the early stage, IL-6 contacts AEC-II and be anti-fibrotic, but later can play a profibrotic role by acting on macrophages (128). Similar dual role has also been proposed for IL-4 (128). Another controversial interleukin is IL-9. Earlier studies have proposed an antifibrotic role for IL-9, while a recent study has shown that antibody-dependent neutralization of IL-9 can reduce silica-induced PF in mice (128, 147). It appears that role of IL-9 is dependent on the type of fibrosis as well as immune cells involved. Another interleukin, IL-22, however, has been linked to negative regulation of PF, as evident from IL-22 mediated suppression of TGF-β1 induced fibroblast-to-myofibroblast transition probably via inhibition of TGF-β1 receptor (TGF-β1R) and subsequent Smad2/3 activation (148). Intranasal instillation of recombinant IL-22 suppressed fibrotic phenotype (148). Thus, depending on their profibrotic or antifibrotic role, ILs could be an important therapeutic candidate against PF.

Humoral growth factors including fibroblast growth factor (FGF), connective tissue growth factor (CTGF) and platelet-derived growth factor (PDGF) also play a crucial role in fibroblast proliferation, trans-differentiation and ECM deposition. Blocking CTGF expression can inhibit collagen production and fibroblast accumulation. PDGF is secreted by cells such as fibroblasts, macrophages, platelets and endothelial cells and its expression is enhanced by TGF-β1. PDGF acts as a mitogen for fibroblasts and plays a significant role in myofibroblast expansion. In addition to being a mitogen for several cells including type I and type II epithelial cells, fibroblasts and airway smooth muscle cells, FGF also stimulates collagen synthesis in myofibroblasts (149).

#### 3.1.4 Toll-like receptors, inflammasomes, damage associated molecular patterns

TLRs are pattern recognition receptors (PPRs), which recognize PAMPs and the endogenous DAMPs (which otherwise remain inaccessible to the immune system). TLR4 has been reported to show profibrotic effect in lipopolysaccharide induced PF (150). TLR4 and its co-receptor adapter myeloid differentiation 2 (MD2) have been implicated in fibroblast-to-myofibroblast transdifferentiation in skin and PF (151). Since High mobility group box 1 protein (HMGB1) is overexpressed in IPF lungs and its antibody mediated neutralization exacerbates BLM-induced fibrosis, role of HMGB1 has been also suggested in PF as a DAMP which eventually stimulates TLR2 and TLR4 in PF (150). TLR2 and TLR4 knockout mice are more sensitive to BLM and radiation-induced pulmonary damage (152). Although unsubstantiated experimentally, HMGB1 and hyaluronan fragments are likely TLR activators in BLM-treated lung (152). BLM itself can act as a direct agonist of TLR2; however, the indirect role of other BLM-induced release of DAMPs is not excluded. Two of the extracellular DAMPs that lead to the formation of extracellular collagen, which causes the ECM stiffness (increase resistance to mechanical stress) as implicated in PF, are tenascin-C and fibronectin-extra domain A (Fn-EDA) which are also known agonists of TLR4 (150).

S100A4, a calcium binding protein and a known ligand of TLR4, has been implicated in PF; S100A4^+^ macrophages are required for PF by activating fibroblast cells (150, 153). Raised serum S100A4 level is as a candidate biomarker for IPF (154). Considering the facts that elevated thrombin activity has been shown in BALF of IPF patients and thrombin-induced degradation of fibrinogen produced proteolytic fragments that can stimulate TLR4 activity, a role of thrombin is likely in the pathogenesis of PF (152). TLR3, which acts as a receptor for endogenous RNA, is considered to have a role in PF in view of the presence of dsRNA in IPF-BALF (152). In 1 out of 8 Caucasian IPF patients, a specific SNP in TLR3 (L412F), which is linked to an aggressive clinical phenotype, has been reported (155). TLR3 deficient mice are susceptible to BLM treatment due to its protective role against generation of type I IFN, an antifibrotic molecule (155). Primary lung fibroblasts derived from TLR3 L412F-variant IPF patients show defective NF-κB and IRF3 activation, cytokine production and type I IFN secretion (155). Stromal cells are the major source of TLR9 in lung tissue derived from IPF patients; in BLM model, TLR9 expression in immune cells triggers antifibrotic effect (155). Moreover, higher expression of TLR9 has been noted in IPF patient-derived fibroblasts triggering fibroblast-to-myofibroblast transdifferentiation as evident from *de novo* synthesis of α-SMA (155). In addition to microbial dsDNA, damaged CpG-rich mitochondrial DNA (mtDNA) released during mitochondrial damage in PF is likely to be another DAMP that triggers TLR9. Extracellular mtDNA is linked to initiation of profibrotic processes (synthesis of α-SMA) in normal human lung fibroblasts in culture (156). Since a number of molecules (such as type I IFN, mtDNA, TLR9), which act upstream and downstream of cyclic GMP-AMP synthase (cGAS)-stimulator of interferon genes (STING), a pathway involved in the regulation of innate and adaptive immunity, contribute to in the pathogenesis of PF, a likely role of cGAS-STING could be suggested in PF (157). Death of epithelial cells at the injured sites is a source of DAMPs as evident from an increased level of a necroptotic regulator RIPK3 in IPF as well as BLM-induced fibrotic lungs (158). Uric acid, released from dead and dying cells, can locally crystallize and form potent DAMPs which trigger fibrosis through NRLP3 inflammasome-activation, MyDD88 and IL-1R1 pathways (and via TLR2 and TLR4 as well) (152, 159). ATP is another DAMP that exerts its action through purinergic P2X receptors (P2X7R) and has been found to be elevated in BALF of IPF patients and in BLM-treated mice (152). In addition, either of the events *i.*e. blocking of P2X7R or exogenous administration of ATPγS aggravates the pathology (152). Receptor for advanced glycation end products (RAGE) is another receptor for DAMPs which is implicated in PF; however, the results are controversial. Both positive and negative regulation of PF has been attributed to RAGE, thus inviting further research on the role of RAGE in PF (152). Although the exact contribution of DAMPs in PF is only partially understood, its interaction with fibroblasts and epithelial and immune cells plays a significant role in the pathogenesis of PF.

Inflammasomes can be classified into four types: absent in melanoma 2 (AIM2), NOD-like receptor protein 1 (NLRP1), NOD-like receptor protein C3 (NLRP3), and NOD-like receptor C4 (NLRC4). NLRP3, which comprises of NLRP3, ASC (apoptosis-associated Speck-like protein containing a CARD) and pro-caspase-1, is most studied in the context of PF. Once the NLRP3 is exogenously or endogenously activated, it recruits ASC which acts as a linker protein connecting NLRP3 with pro-caspase I, thereby forming inflammasome complexes. Activated inflammasome cleaves pro-caspase I to caspase I, which in turn, produces mature IL-1β, IL-18 and IL-33 (160) (**Figure 2B****).** IL-1β and IL-18 exert their effect via their respective receptors (160). On the other hand, IL-33 stimulates Th2 cells to secrete IL-13 and IL-5. The signal that triggers the upstream activation of inflammasomes is via TLRs while it binds to PAMPs or DAMPs, this induces inflammasome expression (160) (**Figure 2B****).** BLM can trigger inflammasomes which lead to PF via IL-1β/IL-1Rs/MyD88/NF-κB signaling axis (160, 161). NLRP3 appears to regulate IL-1β-mediated PF via miR-155 (160, 162). Engineered nanomaterials (ENMs) such as earth oxides, graphene and graphene oxides, fumed silica and high aspect ratio materials can trigger pro-fibrotic milieu in lungs through NLRP3 inflammasome activation and IL-1β production (163). Furthermore, NLRP3 has been also linked to EMT during PF in mechanical stretch-induced PF model (164). Macrophages play a significant role in mechanotransduction (sensing of mechanical stress) via NLRP3 mediated IL-1β production (165). In macrophages, L-plastin (LPL) facilitates the stable interaction of ASC with Pyk2, a component of podosomes, a cell-surface adhesive structure, in turn, activates NLRP3 and downstream IL-1β (160, 165). LPL^-/-^ mice are resistant to BLM-induced lung injury and fibrosis (165). From a therapeutic point of view, in radiation-induced lung injury (RILI) and fibrosis (later phase of RILI), Andrographis, a radioprotective molecule, has been shown to offer protection by suppressing AIM-2 inflammasome-mediated pyroptosis in macrophages. Under radiation, AIM2 translocate, to the nucleus and triggers dsDNA breaks, thereby recruiting ASC to activate caspase-1. This leads to IL-1β and IL-18 production or Gasdermin D cleavage, which in turn, creates membrane pores that release pro-inflammatory cytokines, promoting a profibrotic milieu (166). Another potential therapeutic agent, an alkaloid, lycorine, seems to prevent BLM-induced PF by inactivating NLRP3 inflammasome and pyroptosis PYD domain of ASC (167). Thus, a substantial role of inflammasomes in fibrotic cascades is evident from the current research which makes the inflammasome-components potential therapeutic candidates.

#### 3.1.5 Collectins and complement proteins

Other important innate immune factors involved in pulmonary tissue homeostasis include the collectins (collagen containing calcium-dependent C-type lectins) and the complement proteins. Collectins, SP-A and SP-D, are secreted in the lung alveolar space by alveolar type II epithelial cells and club cells (168). Some of the secreted surfactant proteins, although at a low concentration, can leak into the circulating blood. However, a substantial rise in the serum level of these proteins is observed in IPF patients and can be used as biomarkers for disease progression and mortality (169). A meta-analysis study has revealed that the risk of death in IPF patients is linked with increased SP-A serum level of 39% and a rise in SP-D level by 111% is associated with higher risk, suggesting the importance of SP-A and SP-D in differential diagnosis and prediction of survival in IPF patients (170). A role of SP-D in exacerbating PF complicated with bacterial infection has been documented (171). LPS treatment of macrophages produced IL-12p40, which in turn stimulates the production of IFN-γ and triggers a Th1 response. Through the MAPK/ERK pathway, SP-D/SIRPα signaling inhibits IL-12p40 during bacterial infections, aggravating PF. Reduction in IFN-γ level is correlated with increased expression of profibrotic IL-4/IL-4R, thereby promoting fibroblast activation leading to PF (171). The role of SP-D was also investigated in BLM-treated triple transgenic inducible SP-D mice (iSP-D mice) where SP-D was conditionally expressed under doxycycline (Dox) (172). First of all, the BALF of BLM-treated mice at day 7 showed increased levels of SP-A and SP-D in iSP-D mice on Dox (SP-D on). Additionally, iSP-D mice off Dox (SP-D off) showed more severe PF compared to mice on Dox (SP-D on) after BLM treatment. SP-D genetic deficiency is associated with augmented macrophage-predominant cell infiltration and expression of profibrotic cytokines (such as TGF-β1, platelet-derived growth factor-AA). Moreover, fibrocytes with elevated level of TGF-β1 and CXCR4, which help in fibrocyte recruitment in the lung, are increased in BLM-induced iSP-D mice off DOX (SP-D off). Even in SP-D^-/-^ mice exogenously administered SP-D ameliorated BLM-induced PF (172). A similar result was also observed when SP-A gene deficient mice was challenged with BLM (173). In murine lung epithelial cells (LA-4), TGF-β1 decreased SP-A while exogenous SP-A administration reverted the profibrotic markers which were triggered via TGF-β1 treatment (173).

Complement system is a crucial mediator in the innate immune response (174). Although liver is the main source of complement proteins, several effector and regulatory complement subcomponents can be locally synthesized under inflammatory conditions. In the lung, AEC-II, bronchiolar epithelial cells and alveolar macrophages are local producers of certain complement components. IL-6, IL-1, TNF-α and IFN-γ stimulate complement synthesis in epithelial cells, fibroblasts and polymorphonuclear leukocytes (175). C1q, the first subcomponent of the classical pathway, is positively linked with increased pathology of PF as evident from the amelioration of silica-induced IPF in C1q KO. Single cell RNA sequencing analysis revealed that C1q may be exerting its effect by targeting fibroblast and AEC-II (176). It is also important to note that C1q has been identified as a common hub gene in both IPF and non-small cell cancer (NSCLC), considering that IPF is an independent risk factor for NSCLC (177). In one study with systemic sclerosis associated with PF, anti-C1q autoantibodies have been found in the serum of 20 out of 124 patients, and out of those 20 patients, both circulating immune complexes (CICs) as well as anti-C1q autoantibodies have been observed (**Figure 3Bi****).** The study suggests high level of association of anti-C1q autoantibodies in PF and can also be counted as an important risk factor in PF (178). In a cross-sectional study involving 300 IPF patients and 175 healthy (control) individuals, a relationship was investigated between complement component C3 and gain-of-function MUC5B (mucin 5B, oligomeric mucus/gel-forming) promoter variant (179). The gain-of-function MUC5B promoter variant is linked to a greater risk of IPF. C3 expression was higher in IPF subjects (1.40-fold) overall as well as in IPF patients with high-risk MUC5B promoter genotype. The result was corroborated in a mouse model, where BLM-exposed mice exhibited increased expression of MUC5B protein, while C3-gene-deficient mice were found resistant to BLM-inflicted PF (179). C5 has also been implicated as a pro-fibrotic factor in a BLM-induced PF model, acting via TGF-β1 and MMP-3 (180). Recently, mass spectroscopic analysis of BALF of 22 IPF and 10 healthy controls revealed that the complement and coagulation pathways were one of the most differentially regulated group of proteins in IPF in comparison to control groups (27%). Complement and its cleaved fragments such as C5, C6, C7, C8 and C9 were found significantly upregulated. Complement inhibitory proteins (CIPs) CD46 and CD55 are present on epithelial cells. TGF-β1 can inflict epithelial cell injury thereby downregulating CIPs (180). Lower CIP expression is observed in IPF compared to control. Mechanistically, TGF-β-induced loss of CIPs triggers complement activation that in a feedback loop further downregulates CIPs and stimulates TGF-β1 expression. In IPF patients, 2- and 4-fold increase in C3a level was observed in plasma and lung tissue homogenate, respectively. The level was up by 2-fold for C5a compared to healthy control (180). Similar results were observed *in vitro* when normal primary human small airway epithelial cells were treated with specific doses of TGF-β1, C3a and C5a. A dose dependent decrease of CIPs correlated with increased PARP was evident. Together, these data suggest that TGF-β1 mediated downregulation of CIPs and upregulation of anaphylatoxins such as C3a and C5a cause epithelial injury, which in turn, lead to tissue fibrosis in IPF (180). In patients with severe PF, C1q levels are appear to trend upward. In IPF, C1q expression increases and its methylation status decreases (177). However, it is worth noting that role of complement system in the pathogenesis of PF needs further investigation.

### 3.2 Adaptive immunity

Adaptive immunity comprises of two major arms, T-cell mediated immunity and antibody mediated humoral immunity. A healthy lung usually harbors less CD3, CD4 and CD45RO expressing T cells. Non-fibrotic/non-cancerous lung tissues showed significantly low number of CD3^+^, CD4^+^ (T-helper cells), and CD45RO^+^ (memory cells) cells compared to IPF lung tissues (181). Classically Th1 cytokines, where IL-2, TNF-α and GM-CSF are also produced, stimulate AMs to combat viral and bacterial antigens favoring inflammation. Th2 response, where IL-6, IL-10 and IL-13 are synthesized, dampens Th-1 mediated inflammation. The source of these cytokines are T helper cells, in addition to other lymphocytes and monocytes (182, 183). The severity of fibrosis is tightly controlled by maintaining balance in Th1 and Th2 phenotypes, an imbalance can lead to IPF, where Th-2 response is pro-fibrotic. CD4^+^ T cells play a vital role in the progression of fibrosis and studies on cytokine deficient mice showed that fibrogenesis is closely linked with three Th2 cytokines, IL-4, IL-5 and IL-13 (184). A substantial amount of peripheral CD4^+^ T cells was identified in IPF subjects, expressing MHC class-II and CD40L. These CD4^+^ T cells also showed abnormal clonal proliferation and with high expression of TGF-β1, TNF-α and IL-10 (185). Lung extracts from IPF patients could stimulate autologous CD4^+^ T lymphocytes, a characteristic of cell-mediated immunopathology induced by antigens presented by the diseased tissue.

BLM-induced PF was also found partially hampered in nude mice (athymic mice), suggesting the role of T cells contributing to PF (186, 187). In the pulmonary foci with active disease characterized by fibrosis had significantly large number of cells that expressed CD4, CD8, CD20, CD68, CD80, CCR6, S100, IL-17, TNF-α, and retinoic acid-related orphan receptors (RORs) and less expression of Foxp3, CD56, and CD34 (181). However, the expression of CD8^+^ T cells was almost similar in healthy and IPF tissues. In the subpleural or perivascular region, the CD3^+^ T cells were found surrounding the edges and CD20^+^ B cells in the center. However, the Foxp3^+^ cells were located outside of the inflammatory infiltrates and not in the fibrotic lung region. Th17 lymphocytes were detected in the stromal lung tissue of IPF (181). Immune cells also express SASP markers contributing to immunosenescence (188). T cell senescence is characterized by gradual loss of CD28 markers and a similar phenomenon was also observed in IPF patients (189, 190). Flow cytometry analysis of peripheral blood CD4^+^ T cells of IPF subjects showed presence of CD4^+^CD28^null^ lymphocytes with overexpressed cytotoxic markers such as perforin and granzyme B and less expression of Foxp3, a Treg cell marker. These CD4^+^CD28^null^ T cells also showed infiltration in IPF lungs (190). CD3^+^ T cells from lungs explants from IPF patients were rich in CD8^+^CD28*null* T cells and were found limiting the efficacy of dexamethasone, an immunosuppressant (191). The unusual presence of CD4^+^CD28^null^ T-cells with CD28 down-regulation was associated with IPF manifestations (190).

Fibroblastic foci are small areas which are rich in fibroblasts, myofibroblasts and with enormous amount of ECMs. These areas showed higher level of B cell infiltrations producing pro-fibrotic mediators, antibodies and autoantibodies (192). During several chronic inflammation, tertiary lymphoid structures/organs (TLS), also known as ectopic lymphoid organs, are formed in non-lymphoid organs. TLS are aggregates of immune cells (B cells, T cells and DCs) that are held by intricate meshwork of fibroblasts (193, 194). As mentioned earlier, these ectopic lymphoid structures are formed in the lungs of PF patients (**Figure 3Bi**). A recent study involving early and end-stage IPF subjects showed TLS with CD20^+^ B cells in the lung parenchyma surrounded by a large number of CD4^+^ T cells. These TLS compared to early-stage TLS, the end-stage TLS showed greater positivity toward, activated B cell marker, CD40, suggesting a positive correlation between TLS activation and the disease progression (195). In IPF patients, antigen antibody complexes were found in circulation as well as in the BALF and lung parenchyma (196–198) (**Figure 3Bi****).** In almost 82% of IPF patients, IgG autoantibodies were found that were against cellular antigens (185). Immune complexes that were observed in IPF patients were predominantly IgG (196). In the sera of IPF patients, anti-human cytokeratin-18 (CK18) antibody levels as well as CK18: anti-CK18 antibody complex levels were significantly high when compared to healthy controls (199). In case of progressive IPF, the IL-1α autoantibodies were also generated (200). In the inflammatory foci for B cell homing, C-X-C motif chemokine 13 (CXCL13) is a vital mediator and has been linked with the pathogenesis of IPF, where CXCL13 production was heightened (201). High levels of IgM and IgA were found in the culture supernatant of mononuclear cells isolated from IPF patients, suggesting activation of B cells by B cell growth factor (BCGF) (**Figure 3B**). T lymphocytes from the BALF of IPF patients were able to stimulate proliferation of B cells than those isolated from healthy ones, this could be due to increased production of B cell differentiation factor by T cells (202). When the proportion of memory B cells in the lung and in the blood of IPF patients were investigated, IgA expressing B cells were significantly higher over memory B cells with other Ig subclasses. In the same cohort, large number of TLS were found in the lungs with strong IgA staining and had higher IgA anti-nuclear autoantibodies in the plasma of IPF patients. These patients also showed high expression of Bruton’s tyrosine kinase, a protein which has a strong association with autoimmune disorders (203).

## 4 Clinical trails and disease models

Despite the continuous effort, no treatment has emerged to cure PF. Moreover, along with the other risk factors, in the post-COVID scenario, the situation has worsened. Notably, one of the most significant long-term complications of COVID-19 is PF. The situation is grave as asymptomatic patients are also potentially susceptible to PF (204). Thus, in years to come the burden of PF is likely to be high. Therefore, there is a desperate need for an effective non-invasive therapy. Until 2011, the only approved medical treatment available for PF were oxygen supplementation, lung transplantation and pulmonary rehabilitation (205). Since then, two FDA drugs have been approved, perfenidone and nintedanib, to treat PF (1). But these drugs can only slow down the disease progression without having full cure. Thus, an effective non-invasive therapeutic intervention is warranted. However, within the last decade, a number of drugs or treatment regimen have reached the stage of clinical trial and their published clinical data listed in the **Table 1** (6, 206). A number of clinical trials are currently underway; many of them have been already completed. Interestingly, a substantial number of trials have been terminated midway. Several trials which were short-term, conducted for 12 weeks, are limited in commenting on the long-term use of these drugs. However, several drugs have shown promising results at the preliminary stages of clinical trials. Finding a pharmacological solution of the disease is still an enigma as most of the drugs effective in preclinical studies ultimately fail to turn out to be an effective prescribable drug. There are several reasons for this. One of the challenges could be the gap of translatability that exists between preclinical drug testing and human trials. To enhance the translatability the preclinical model systems shall be carefully designed so that they mimic as close as possible to the pathological condition. Till date, the existing PF models include use of chemical agents such as bleomycin, asbestos, silica, profibrotic cytokines such as TGF-β, TNF-α, IL-1β, IL-13, acid-instillation, radiation, age-induced and inducing transgene such as mutant surfactant protein C gene (SFTPC) (207, 208). These models using a single agent or targeting single gene fails to mimic the human PF condition, which is multifactorial. For example, BLM the most commonly used agent to induce PF in animal models, resembles acute lung injury to some extent but fails to mimic the gradual irreversible progression of IPF in human, as BLM-induced fibrosis in mice is partially reversible (209). Additionally, in mice, tail vein mediated delivery of BLM has been shown to be more effective in developing PF that resembles human IPF condition compared to intraperitoneal and intratracheal instillation, suggesting that route of delivery can also determine the variation in fibrogenesis (210). Moreover, selection of the right animal strain is also important. Although spontaneous fibrosis has been observed in some animals including dogs, cats, horse and chickens, the rodents are still the most effective model for PF with 95% genetic similarity to human. Notably, the strain selection significantly controls the susceptibility of fibrosis in an organ-specific manner that ultimately determines the strain specific response to the disease. For example, BALB/c mice show resistance to PF while C57BL/6J are susceptible (207). Interestingly, three fold lower level of TGF-β1 expression was observed in BALB/cBy mice compared to C57BL/6J (211). One study has shown that T-bet pathway of CD4^+^ T cells can render this resistance to BLM-mediated PF in BALB/c mice (212). In this context, *in vitro* models are gaining importance. Although they are the simplified representation of the actual disease condition, they can be important in executing focused studies on identifying particular cellular and molecular pathways that promote disease progression (208). Recently, 2D and 3D cultures obtained from normal and diseased individuals have gained immense attention for unleashing the underlying mechanism with better clarity. Moreover, microfluidics has also contributed to our understanding of the physiology of the respiratory systems *ex vivo* (208). Thus, a successful clinical trial should be based on, finding perfect strategies for selecting models of PF at the preclinical stages.

**Table 1:** Drugs under clinical trials against IPF.

Name of the Drug	Mode of action/Dose/Route	Trial identifier	Clinical Phase	Study design	Primary outcome	Status	Reference
N115	Depletion of inflammatory agents in the lungs and nasal route and permit nitric oxide to reach bronchi and rise bronchodilation; nasal spray	NCT06037408	Phase III	Randomized, double-blind and placebo-controlled	Percentage variation in coughing incidence per day from baseline to day 21	Completed	(213)
GLPG1690/ziritaxestat	Autotaxin inhibitor; 600 mg once daily; oral	NCT03733444 NCT03711162	Phase III	Randomized, double-blind, parallel group, placebo-controlled	Rate of decrease in FVC from baseline to 52^nd^ week of treatment	Terminated	(214, 215) NCT03733444 NCT03711162
Pamrevlumab	Human monoclonal antibody against CTGF; intravenous	NCT04419558	Phase III	Randomized, double-blind, placebo-controlled	Proportion of patient with disease development (absolute FVC percentage expected at least 10% decline or death) from baseline to 52^nd^ week of treatment.	Terminated	(214) NCT04419558
PBI-4050	Low molecular weight compound against GPR40 and GPR84, against fibroblast to myofibroblast differentiation, reduces CTGF and IL-6; oral	NCT02538536	Phase II	Single-arm, open-label	Significant decrease in FVC from baseline to 12^th^ week of treatment	Completed	(215, 216)
TD139	Galectin-3 inhibitor; 3 mg/10 mg once daily; inhaled	NCT03832946	Phase IIb	Randomized, double-blind, parallel, placebo-controlled	Decrease in FVC (mL) from baseline to 52^nd^ week of treatment	Completed	(214) NCT03832946
PLN-74809	Dual selective inhibitor of α_v_β_1_/α_v_β_6_ inhibitor; oral	NCT04396756	Phase IIa	Randomized, double-blind, dose-ranging, placebo-controlled	Number of individuals with treatment related AEs and test abnormalities	Completed	(214) NCT04396756
STX-100/BG00011	Humanized monoclonal antibody, α_v_β_6_ inhibitor, multiple escalating doses; subcutaneous	NCT03573505	Phase IIb	Randomized, double-blind, placebo-controlled	Change in FVC between baseline and 52^nd^ week	Terminated	(215, 217)
IDL-2965	α_v_β_1,_ α_v_β_3_ and α_v_β_6_ selective inhibitor, once daily; oral	NCT03949530	Phase I	Randomized, double-blind, placebo-controlled; single/multiple dose study	Emergence of adverse outcomes in course of treatment	Terminated	(215) NCT03949530
Pamrevlumab/FG3019	Completely human monoclonal antibody against CTGF; 30 mg/kg every 3 week; intravenous	NCT03955146	Phase III	Randomized, double-blind, placebo-controlled	Alteration in FVC (litres) from baseline to 52^nd^ week	Terminated	(214, 215) NCT03955146
PLN-74809	α_v_β_1_ and α_v_β_6_ selective inhibitor; 40 mg in everyday; oral	NCT04072315	Phase IIa	Randomized, sequential assignment	Number of individuals with altered α_v_β_6_ receptor occupancy from baseline as analysed by PET	Completed	(214) NCT04072315
Treprostinil	Inhibition of PDE-5; inhaled	NCT02630316	Phase II PhaseIII	Randomized, double-blind, placebo-controlled and parallel-group	Alteration in the peak 6MWD at week 16	Completed	(213, 218) NCT02630316
Saracatinib	Highly selective Src tyrosine kinase family inhibitor; oral	NCT04598919	Phase Ib/IIa	Randomized, double-blind, parallel design, placebo-controlled	Safety, pharmacokinetics, pharmacodynamics, tolerability, efficacy (as determined by alteration in FVC from baseline to 24^th^ week of tretment	Active not recruiting	(214) NCT04598919
BI1015550	Phosphodiesterase-5- inhibitor, multiple rising doses; oral	NCT03422068	Phase I	Randomized, double-blind, placebo-controlled	Proportion of individuals experiencing drug associated adverse events (AEs)	Completed	(215) NCT03422068
rhPTX-2/PRM-151	TGF-β1 modulator; intravenous	NCT04552899	Phase III	Randomized, double-blind, placebo-controlled	Thorough change in FVC (mL) from baseline to 52^nd^ week	Terminated	(214) NCT04552899
TD139	Galectin-3 inhibitor; inhaled	NCT02257177	Phase I/IIa	Randomized, double-blind, multicenter, placebo-controlled	Number of individuals reporting AEs from the date of first dose, until 30 days post first dose	Completed	(215, 219)
BMS-986278	LPA antagonist; oral	NCT04308681	Phase II	Randomized, double-blind, placebo-controlled	Rate of change in percentage predicted FVC from baseline to week 26	Completed	(214) NCT04308681
IW001	Antibody against type V collagen; oral	NCT01199887	Phase I	Open-label, multicenter study	Designed to determine the safety, tolerability, biological and clinical effects of three different doses of IW001 in IPF patients who were anti-col(V) Ab+. While patients in the lowest-dose cohort experienced a downfall in FVC comparable to that observed in placebo arms of previous IPF trials. The highest-dose cohort showed a trend toward stabilization of FVC and reduced binding of C1q to anti-Col V antibodies consistent with an IW001-induced effect on anti-Col V antibody binding and activity, alteration from baseline to 24^th^ week of treatment in FVC.	Completed	(215, 220)
GLPG1205	GPR84 antagonist; 100 mg once daily; oral	NCT03725852	Phase II	Randomized, double-blind, placebo-controlled	Change in FVC from baseline to 26^th^ week	Completed	(214) NCT03725852
Pamrevlumab	Against CTGF; 30 mg/kg or every 3 weeks over 48 weeks in placebo; intravenous	NCT01890265	Phase II	Randomized, double-blind, placebo-controlled	As compared to placebo, the decrease in FVC drop at 48 weeks in the treatment group	Completed	(213, 221)
rhPTX-2/PRM-151	TGF-β1 modulator; intravenous	NCT04594707	Phase III	Open-label extension study	Occurance of severe adverse effects (AEs) and infusion related activities; percentage of individuals permanently discontinuing study treatment beacuse of AEs	Terminated	(214) NCT04594707
GSK2126458/Omipalisib	PI3Ks/mTOR inhibitor, Omipalisib 0.25 mg/1 mg/2 mg twice daily; oral	NCT01725139	Phase I	Randomized, double-blind, placebo-controlled, repeat dose escalation	PD endpoints pAKT/AKT in BAL cells and pletelet-rich plasma; AUC in blood for GSK2126458; Cmax in blood for GSK2126458; pre-dose concentration at the completion of the dosing interval in blood for GSK2126458; concentration of GSK2126458 in BAL fluid	Completed	(215) NCT01725139
KD025/SLx-2119	ROCK2 inhibitor; 400 mg once daily; oral	NCT02688647	Phase II	Open label	Change in FVC from baseline to 24^th^ week of treatment; number of individuals experiencing AEs	Completed	(214) NCT02688647
VAY736/ianalumab	IgG1 monoclonal antibody against BAFF receptor; 300 mg or a placebo given every four weeks for 48 weeks; subcutaneous	NCT03287414	Phase II	Randomized, sponsor-blinded, placebo-controlled	Alteration from baseline to 48^th^ week in FVC	Terminated	(214, 222) NCT03287414
ND-L02-s0201/BMS-986263	HSP47 inhibitor; dose level 1/dose level 2, every two weeks; intravenous	NCT03538301	Phase II	Randomized, double-blind, placebo-controlled	Number of individuals with treatment-associated AEs from baseline to 24^th^ week	Completed	(214, 215) NCT03538301
Dasatinib + Quercetin	Elimination of senescent cells, (100 mg/d) + Quercetin (1250 mg/d), three doses given over three consecutive days in three consecutive weeks; oral	NCT02874989	Phase I	Randomized (some patients are randomized to placebo or study drug while others go into open label)	Percentage of pro-inflammatory expressing cells in skin biopsy obtained both at baseline and 4^th^ week	Completed	(214, 215) NCT02874989
CC-90001	Selective JNK inhibitor; oral	NCT03142191	Phase II	Randomized, double-blind, placebo-controlled	Alteration in percentage predicted FVC from baseline to week 24	Terminated	(214) NCT03142191
C21	Angiotensin type 2 receptor agonist; oral	NCT04533022	Phase II	Open-label, single-arm	Nature and frequency of AEs occurring over the trail period	Completed	(214) NCT04533022
Human autologous lung stem cells	Immunomodulatory, anti-proliferative, and anti-inflammatory; Lung spheroid stem cells 100/200 million; intravenous	NCT04262167	Phase I	Randomized, open label	Number of patients with both AEs and severe AEs	Suspended	(214) (215) NCT04262167
GKT137831/Setanaxib	NOX1 and NOX4 inhibitor; 400 mg twice daily; oral	NCT03865927	Phase II	Randomized, double-blind, placebo-controlled	Oxidative stress related surrogate biomarker by mass spectrometry from baseline to week 24	Completed	(214, 215) NCT03865927
Human autologous lung stem cells	Immunomodulatory, anti-proliferative, and anti-inflammatory; injected by bronchoscopy	NCT02745184	Phase I/Phase II	Open label	Change in FVC from baseline to week 48	Completed	(214) NCT02745184
Jaktinib Dihydrochloride Monohydrate	JAK 1, JAK 2 and JAK 3 inhibitor; oral	NCT04312594	Phase II	Randomized, double-blind, placebo-controlled	Alteration in FVC from baseline to week 24	Completed	(214) NCT04312594
TD-1058	Undefined; inhaled	NCT04589260	Phase I	Randomized, double-blind, parallel-group, placebo-controlled	Number and severity of treatment emergent AEs	Terminated	(214) NCT04589260
TRK-250	Nucleic acid suppressing expression of TGF-β1 protein at gene expression level; single and multiple inhaled dose (4 weeks); inhaled	NCT03727802	Phase I	Randomized, double-blind, placebo-controlled, single and multiple dose	Incidence and severity of AEs up to 7 days after last dose	Completed	(214, 215) NCT03727802
INS018 _055 (ISM001-055)	Inhibition of TNIK; oral	NCT05938920	Phase IIa	Randomized, double-blind, placebo-controlled	Proportion of patients have at least 1 TEAE	Completed	(213)

CTGF, connective tissue growth factor; FVC, forced vital capacity; AE, adverse event; GPR40, G Protein-coupled Receptor 40; GPR84, G Protein-coupled Receptor 84; IL, interleukin; PET, positron emission tomography; PDE-5, phosphodiesterase-5; 6MWD, 6 minutes walking test; LPA, lysophosphatidic acid; PI3K, phosphatidylinositol 3-kinases; mTOR; mammalian target of rapamycin; pAKT, phosphorylated AKT; PD, pharmacodynamic; BAL, bronchoalveolar lavage; GSK, glycogen Synthase Kinase; AUC, area under the curve; Cmax, maximum observed concentrations; ROCK, Rho-associated coiled-coil kinase; BAFF, B-cell activating factor; HSP, heat-Shock Protein; JNK, c-Jun N-terminal kinase; JAK, Janus kinase; ppFVC, percent predicted forced vital capacity; NOX, nicotinamide adenine dinucleotide phosphate (NADPH) oxidase; TGF-β1, transforming growth factor β1; TNIK, Traf2 and Nck-interacting kinase; TEAE, treatment emergent adverse events.

## 5 Conclusion

Owing to the rapid rate of increase of pollutants, rise in the percentage of global population aged 65 or above, the silent progression of long COVID related PF without early manifestation and the dearth of effective therapeutic intervention, a respiratory disease related imminent catastrophe can be predicted. Thus, understanding the disease pathology with all molecular and cellular intricacies is quintessential. In this respect, growing evidence based on clinical-translational studies and targeted molecular and cellular therapies indicate an intricate interplay of the immune and the non-immune cells in modulating the lung microenvironment that leads to the establishment of pulmonary fibrosis. In PF, a persistent inflammation causing collagen rearrangement in the lungs exists which is probably irreversible; however, the dysregulated inflammation aggravating the disease can possibly be controlled, if diagnosed early. At present, the treatments for IPF are very limited, and the need of the hour is to find inflammatory biomarkers for precise therapeutic approaches.
